# Ion Channel Drugs Suppress Cancer Phenotype in NG108-15 and U87 Cells: Toward Novel Electroceuticals for Glioblastoma

**DOI:** 10.3390/cancers14061499

**Published:** 2022-03-15

**Authors:** Juanita Mathews, Franz Kuchling, David Baez-Nieto, Miranda Diberardinis, Jen Q. Pan, Michael Levin

**Affiliations:** 1 Allen Discovery Center at Tufts University, Medford, MA 02155, USA; juanita.mathews@tufts.edu (J.M.); franz.kuchling@tufts.edu (F.K.); midiberardinis@comcast.net (M.D.); 2 Stanley Center of Psychiatric Research, Broad Institute of Harvard and MIT, Cambridge, MA 02142, USA; dbaez@broadinstitute.org (D.B.-N.); jpan@broadinstitute.org (J.Q.P.); 3 Wyss Institute for Biologically Inspired Engineering, Harvard University, Boston, MA 02115, USA

**Keywords:** cancer, glioblastoma, NG108-15, U89, ion channel, blockers, openers

## Abstract

**Simple Summary:**

Glioblastoma is a rapidly progressing brain cancer that is very difficult to treat. Given that many aspects of cell and tissue behavior are controlled by electric signaling, we sought to test whether drugs that target ion channel proteins might be effective at controlling the spread and functionality of glioblastoma cells in culture. Testing aspects of cell growth and physiology, we show that several novel combinations of ion channel drugs, which are already approved in human patients for other purposes, are highly effective against two types of glioblastoma cells. This facilitates the development of new strategies to address cancer by repurposing the large class of ion channel drugs against cancer.

**Abstract:**

Glioblastoma is a lethal brain cancer that commonly recurs after tumor resection and chemotherapy treatment. Depolarized resting membrane potentials and an acidic intertumoral extracellular pH have been associated with a proliferative state and drug resistance, suggesting that forced hyperpolarization and disruption of proton pumps in the plasma membrane could be a successful strategy for targeting glioblastoma overgrowth. We screened 47 compounds and compound combinations, most of which were ion-modulating, at different concentrations in the NG108-15 rodent neuroblastoma/glioma cell line. A subset of these were tested in the U87 human glioblastoma cell line. A FUCCI cell cycle reporter was stably integrated into both cell lines to monitor proliferation and cell cycle response. Immunocytochemistry, electrophysiology, and a panel of physiological dyes reporting voltage, calcium, and pH were used to characterize responses. The most effective treatments on proliferation in U87 cells were combinations of NS1643 and pantoprazole; retigabine and pantoprazole; and pantoprazole or NS1643 with temozolomide. Marker analysis and physiological dye signatures suggest that exposure to bioelectric drugs significantly reduces proliferation, makes the cells senescent, and promotes differentiation. These results, along with the observed low toxicity in human neurons, show the high efficacy of electroceuticals utilizing combinations of repurposed FDA approved drugs.

## 1. Introduction

Glioblastoma (GBM) is a cancer of the brain that has a high mortality rate and a short median survivability time of around 14 to 15 months [1]. Prognosis is particularly poor for glioblastoma patients due to the failure of standard interventions in preventing tumor re-growth. Currently, the standard treatment for GBM is resection of the main tumor followed by radiation and temozolomide (TMZ) administration [2]. However, reoccurrence of GBM is common (90%) and usually arises from the area immediately next to the resected area [3,4]. Thus, the invasiveness and hypermigratory nature of these residual cancer cells make resection alone ineffective; additional interventions that prevent the proliferation of these cells are needed to complement current methodologies.

Targeting specific cell types in glioblastoma has proven difficult due to their heterogeneous nature. Although the WHO classifies glioblastoma as a grade IV astrocytoma, GBM likely arises from a combination of both neural stem cells (NSCs) in the subventricular zone (SVZ) and oligodendrocyte progenitor cells (OPCs) that differentiate from those NSCs [5,6,7,8]. Further phenotypic blurring occurs when oncogenic gene expressions lead to an astrocytic or oligodendroglial appearance [9]. In Vivo, OPC-like cells are enriched at the periphery of GBM tumors. These cells and microglia serve as a source of growth factors that give GBM cells more stemness, facilitate growth and proliferation, and create a supportive tumor microenvironment [10]. In addition, GBM cells in the border niche have upregulated levels of transcripts for drug efflux transporters and radioresistance [10], which may underlie resistance to TMZ treatment during radiation [10]. Thus, the proliferative capability of these border areas and resistance to drug therapy and radiation likely explain the high reoccurrence of GBM after tumor resection and chemotherapy. Treatment strategies that target the proliferative capability of GBM and bypass typical drug efflux transporters could potentially increase the survivability of this fatal disease.

Differentiation as a treatment strategy for GBM has recently been explored by several labs. Instead of killing the cancer cells, this method attempts to remediate the cancer cells so that they no longer proliferate or invade [11,12,13]. Studies have successfully used all-trans-retinoic acid (ATRA), molecules that upregulate cAMP production, bone morphogenic protein 4 (BMP4), and most recently Pam3CSK4, a synthetic Toll-like receptor 2 (TLR2) ligand to differentiate GBM cells [14,15,16,17,18,19]. However, retinoid treatment can be prone to resistance effects, and some of the other methods suffer from off-target effects that can require invasive targeted treatment strategies and limit in vivo use [20].

Alongside canonical biochemical factors and biomechanical forces, cell and tissue-level order are now known to be regulated by bioelectrical signaling [12,21]. The transmembrane voltage (V_mem_) of cells is regulated by ion channels, which are not only important targets in embryonic channelopathies [22,23,24] but also increasingly seen as cancer targets [22,25,26,27,28,29,30,31,32,33,34,35,36]. Overall, adult non-proliferative cells tend to have hyperpolarized membrane potentials (around −40 mV to −90 mV), whereas stem cells, embryonic cells, and other highly proliferative cells are much more depolarized (around −5 mV to −40 mV) [22,37]. Cancer cell V_mem_ tends to have much more depolarized membrane potentials than their non-cancerous equivalents [24,37]. Indeed, transformed cells can be detected in vivo in animal models using voltage-sensitive fluorescent dyes based on their abnormal bioelectric signature [38]. Importantly, the resting membrane potential is not only a marker but is functionally instructive for cell behavior. Classic work by Cone showed that V_mem_ is an important regulator of proliferation in terminally differentiated cells or cancer [39,40,41], and this has been confirmed in recent work linking depolarization with a plastic, undifferentiated, highly proliferative state. For example, treatment of mesenchymal stem cells with depolarizing drugs inhibited their differentiation into adipocytes or osteoblasts, suggesting that hyperpolarization is required for differentiation [42,43,44,45,46]. Importantly, in vivo experiments in *Xenopus* tadpoles showed that co-expression of hyperpolarizing ion channels, or optogenetic activation of channels with human oncogenes, can prevent the tumors induced by oncogene expression alone [34,38,47,48]. In mammalian models, several recent studies have shown that ion channelopathies that affect resting membrane potential are present in many cancers and play a key role in cell proliferation, progression through the cell cycle, and metastasis [25,29,31,36,49,50,51,52,53,54,55,56].

The location of many ion channels in the plasma membrane also makes them attractive therapeutics targets. Agonists or antagonists used to modulate V_mem_ can be chosen for binding sites on the outer side of the membrane, bypassing the challenges posed by increased drug efflux transporters in glioblastoma stem cells (GSCs). Furthermore, ion channel transcripts have been shown to be upregulated in GSCs, including *SCN8A*, which encodes a sodium channel, *KCNB1*, which encodes a voltage-gated potassium channel, and *GRIA3*, which encodes an ionotropic glutamate receptor that is non-selective for monovalent cations [53]. Other studies have found multiple changes in a variety of potassium channels in GBM, including an increase in BK channel expression, a decrease in hERG1, and a decrease in Kir4.1 [57,58]. In addition, intracellular alkalization induced by dysfunction in proton transportation has been shown to increase drug resistance in GBM and contributes to extracellular acidification, which provides GSCs with an optimized niche and facilitates the release of large oncosomes, a type of extracellular vesicle that can transform neighboring cells [59,60,61]. Thus, due to the location and importance of these channels in cancer cell proliferation, migration, and metastasis, ion channels provide an optimal target for the development of new GBM treatments.

A large catalogue of FDA approved pharmaceuticals that modulate ion channels exist, creating an opportunity to directly assess the role of V_mem_ in cancer progression. To discover effective interventions for glioblastoma using known small molecule modulators of the bioelectric state, we first used the cell line, NG108-15, to screen for V_mem_-modifying compounds that could potentially promote differentiation in glioblastoma cells [62]. NG108-15 cells are a hybrid formed from mouse N18TG2 neuroblastoma cells with rat C6-BU-1 glioma cells and is a popular model system in neuronal differentiation studies and shows cancer stem cell characteristics. This line was chosen due to its extensive use in determining the factors necessary for neuronal differentiation, its close resemblance to NSCs and GSCs, and its well-characterized membrane properties and electrophysiology [63,64,65]. Our goal was to find compounds that would safely abrogate the proliferative potential in NG108-15 cells under high serum conditions, normally prohibitive for differentiation and representative of the high plasma protein content found in vivo, revealing robust candidates for subsequent assessment in the U87 (ATCC) human glioblastoma line, and for future testing in patient-derived GSCs [66,67,68,69].

To screen candidate drugs for anti-cancer activity, we first created new cell lines as resources for future studies in this field: an NG108-15 and U87 stable line expressing a Fluorescence Ubiquitin Cell Cycle Indicator (FUCCI) [70] and a cell membrane mTurquoise tag for revealing whole-cell morphology. Automated electrophysiology was used to determine the overall plasma membrane potential in NG108-15 cells in the presence of the drug compound treatment. For U87 cells, we used dyes to determine cytoplasmic calcium levels, internal pH (pHi), transmembrane potential and lysosomal pH to gain comprehensive profiling of the drugs’ effects on cell physiology. Finally, a beta-galactosidase stain and immunocytochemistry were used to analyze senescence, cell cycle inhibitor levels, and differentiation status of treated NG108-15 and U87 cells. In addition, we used human neurons to perform a Live/Dead assay and a senescence assay to identify treatments that were toxic to non-cancerous cells. We identified several combinations of compounds that strongly and specifically reduce proliferation and promote induction of differentiation, demonstrating that this class of electroceuticals, most of which are already FDA approved for human use in other conditions, are good candidates for cancer remediation in GBM.

## 2. Materials and Methods

### 2.1. Reagents

Stocks of Rapamycin (R8781, Sigma, St. Louis, MO, USA), Retigabine (SML0325, Sigma), Minoxidil (M4145, Sigma), NS1643 (sc-2041353, Santa Cruz Biotechnology, Dallas, TX, USA), Lamotrigine (L3791, Sigma), Zolmitriptan (SML0248, Sigma), Cariporide (SML1360, Sigma), Topiramate (T0575, Sigma), Pantoprazole Sodium Hydrate (P0021, Sigma), Fenofibrate (F6020, Sigma), Acetazolamide (A6011, Sigma), Quercetin (Q4951, Sigma), Temozolomide (2706, Tocris, Bristol, UK), Dexamethasone (D4902, Sigma), ONO-RS-082 (O0766, Sigma), Topotecan hydrochloride (4562, Tocris), CKD 602 (5125, Tocris), (Z)-4-hydroxytamoxifen (3412, Tocris), Lansoprazole (2582, Tocris), Chlorzoxazone (C4397, Sigma), and Sodium Butyrate (B5887, Sigma), were made at 1000× concentration in DMSO. Stocks of Gabapentin (G154, Sigma) and Cisplatin (232120, EMD Millipore, Burlington, MA, USA) were made in water at 1000×. Dibutyryl cAMP sodium salt (D0627, Sigma) was the only compound dissolved directly in cell culture media at 1 mM concentration.

### 2.2. Cell Culture

NG108-15 cells (ATCC), passage 7–10 were cultured in growth media containing DMEM medium with high glucose and no phenol red or sodium pyruvate (31053-028, ThermoFisher, Waltham, MA, USA), which was supplemented with 2 mM Glutamax, 10% FBS, HAT supplement, and 100 U/mL penicillin/streptomycin. Cells were disassociated with Accutase and passaged when 70% confluent and maintained at 37 °C with 5% CO_2_. The media were changed every two days. For live cell assays, plates were coated with 1/50 dilution of no phenol red growth factor reduced (GFR) Matrigel (356231, Corning, NY, USA) in imaging media made of Fluorobrite DMEM (A1896701, ThermoFisher) with no added supplements. Cell culture medium was switched to Fluorobrite DMEM with 2 mM Glutamax, 10% FBS, HAT supplement, and 100 U/mL penicillin/streptomycin for all live cell imaging experiments, except for a cAMP with rapamycin treatment which was conducted in the exact same media except with 1% FBS. All drug screens, including controls, were performed using 0.02% DMSO in the high serum Fluorobrite DMEM.

U87 (ATCC) cells were cultured under the same conditions as the NG108-15 cells minus the HAT supplement and used at passage 7–10 for experiments. All cells plated for antibody staining and senescence assays were cultured on polyethylenimine (181978, Sigma) coated plates at 25 µg/mL in 150 mM NaCl solution for 1 h at room temperature followed by a rinse of PBS and an additional coating of 1/50 Matrigel as described above.

Human neuronal cells were differentiated from human induced neural stem cells (hiNSCs) (passage 7–10), a generous gift from David Kaplan, as described previously [71]. Briefly, hiNSCs were grown on mouse embryonic fibroblast (MEF) feeder cells until ready to differentiate into neurons. Cells were then seeded on Poly-D-Lysine (A3890401, ThermoFisher) and laminin (L2020, Sigma) coated 96-well plates at a density of 128,000 cells/mL. Cells were differentiated in Neurobasal (12348017, ThermoFisher) media supplemented with 2% B27 (17504044, ThermoFisher), 1% Glutamax, and 1% antibiotic-antimycotic for 7 days with media changes every 2 days. At the end of the 7 days the treatments were added in the differentiation media for 3 days.

### 2.3. Molecular Biology

The ES-FUCCI construct containing a hygromycin resistance cassette was subcloned using XmnI and SalI from the plasmid ES-FUCCI, which was a gift from Pierre Neveu (Addgene (Watertown, MA, USA) plasmid #62451; http://n2t.net/addgene:62451 (9 March 2022); RRID:Addgene_62451) [70]. The CAG pPalmitoyl-mTurquoise2 construct was subcloned from the plasmid pPalmitoyl-mTurquoise2 using BamHI and NotI, which was a gift from Dorus Gadella (Addgene plasmid #36209; http://n2t.net/addgene:36209 (9 March 2022); RRID:Addgene_36209) [72]. All subcloned fragments were cloned into a pENTR1A plasmid with a CAG promoter and multiple cloning site (MCS) followed by an SV40 poly(A) using the same sites as were used in excising the fragment from the parent plasmid. In the case of ES-FUCCI, the CAG promoter, MCS, and poly(A) were removed from the pENTR1A plasmid with SpeI, which was blunted, and SalI prior to ligation with the fragment. The resulting pENTR1A ES-FUCCI was then Gateway LR clonased (11791020, ThermoFisher) into the hyperactive piggyBac transposase-based, helper-independent, and self-inactivating delivery system, pmhyGENIE-3, a gift from Stefan Moisyadi [73,74]. The pENTR1A CAG pPalmitoyl-mTurquoise2 was cloned into a pmhyGENIE-3 containing a neomycin resistance gene in the backbone. The resulting plasmids, HypG3 Hygro ES-FUCCI and HypG3 NeoBB CAG pPalmitoyl-mTurquoise2 were used for subsequent transfections.

### 2.4. Generation of Stable Lines

All transgenic cell lines were made by transfecting cells at 30% confluence with 500 ng of appropriate HypG3 plasmid via 1 µL of lipofectamine 3000 (L3000008, ThermoFisher) per well of 24-well plate containing 500 µL of culture medium. The reagent was removed after 24 h, and fresh culture medium was added. Cells were allowed to recover for 24 h prior to selection with 1000 µg/mL G418 or 200 µg/mL hygromycin. After selection, cells were serially diluted into 96-well plates, and single colony clones were expanded. Clones showing robust growth and strong expression were chosen for subsequent experiments.

### 2.5. Growth and FUCCI Assays

Both cell lines were plated at 5000 cells per mL into black-walled flat-bottom 96-well plates coated with 1/50 dilution of Matrigel. Images of cells were taken on Day 0 and every subsequent day for 10 days using a Zeiss Axio1 fitted with an on-stage incubator and kept at 37 °C with 5% CO_2_. A 5× objective was used along with filters for YFP, RFP, and CFP, and careful calibration of each plate was undertaken to ensure that the same fields of view were imaged each day. Drugs were added after the initial images were taken and then changed every two days until day 6. For recovery experiments, after imaging on day 6, all drugs were removed, and cells were put into imaging media without any added drugs. This media was changed every two days until day 10. Analysis of growth and snap shots of FUCCI reporters over the 6 days or 10 days was conducted using a CellProfiler [75] pipeline designed by the Broad. The fractional difference in cell number was calculated by dividing the total number of cells each day by the total number of cells from day 0. The ratio of cells in each cell cycle stage every day was determined by counting all the nuclei of one color and dividing it by the total number of nuclei. Blind quality control was performed on all the images to make sure that any fibers or image artifacts were not incorrectly counted by the program.

### 2.6. Antibody Staining, BrdU and Senescence Assays

NG108-15 cells were plated on PEI and Matrigel-coated plates at 15,000 or 30,000 cells/mL, U87 cells at 10,000 cells/mL and grown in imaging media for 6 days with drug treatment. Fresh media was added every 2 days. On day 6, cells were fixed with 4% formaldehyde in PBS for 30 min for all antibody staining, except for BrdU which was fixed for 15 min, and for the senescence assay, which was fixed for 20 min using the Senescence Beta-Galactosidase Staining Kit (9860, CellSignaling, Danvers, MA, USA) and thereafter stained according to manufacturer’s protocol. After fixing, cells were washed twice with PBS and were permeabilized in PBS with 0.3% Triton-X for 15 min, then washed again twice with PBS. The cells for the BrdU assay were additionally treated with 1N HCl for 10 min on ice, followed by treatment with 2N HCl for 50 min at room temp. Cells were then blocked with standard blocking buffer comprised of TBS containing 10% goat serum, 0.1% BSA, and with 0.05% Tween 20 or without detergent for antibodies against phosphorylated proteins or monoclonal antibodies except for Anti-YAP and Anti-BrdU staining, which were followed according to manufacturer’s protocol. Primary antibodies were added at the following concentrations: 1:250 anti-S100 beta (GTX129573, Genetex, Irvine, CA, USA), 1:500 anti-GFAP (AB5804, EMD-Millipore, Burlington, MA, USA), 1:300 anti-SOX10 (ab155279, Abcam, Cambridge, UK), 1:100 MAP2 (4542, CellSignaling), 1:400 anti-cleaved caspase 3 (9661, CellSignaling), 1:300 anti-Cx43 (STJ2411, St. John’s Laboratory, London, UK), 1:800 anti-p27^Kip1^ (3698, CellSignaling), 1:150 anti-BrdU (5292, CellSignaling), 1:125 anti-LC3B (2775, CellSignaling), 8 µg/mL anti-O4 (MAB1326, R&D Systems, Minneapolis, MN, USA), 1:1000 anti-TH (RPCA-TH, EnCor, Gainesville, FL, USA), 1:4000 anti-NSE (RPCA-NSE, EnCor), 1:4000 anti-NFM (RPCA-NF-M, EnCor), 0.5 µg/mL anti-TujI (801202, BioLegend, San Diego, CA, USA), 1:400 anti-NF-KB p65 (8242, CellSignaling), 1:125 anti-phospho CREB (05-807, EMD-Millipore), 1:250 anti-vimentin (ab92547, Abcam), 1:125 anti-YAP (14074, CellSignaling). Antibodies were diluted in their respective blocking buffer and incubated overnight at 4 °C. The next day, cells were washed with TBS-T or TBS for 3× for 5 min each, and secondary antibody was added as follows: 1:1000 donkey anti-mouse 647 (A-31571, ThermoFisher) or 1:1000 donkey anti-rabbit 647 (A-31573, ThermoFisher). Each was diluted in blocking buffer along with 2.5 µg/mL Hoechst 33342 and incubated for 1 h at room temperature. Cells were then washed again with TBS-T or TBS for 3× for 5 min each and covered with Gelvatol. Cells were imaged with an EVOS M7000 system at 20X, with at least 50% of each well in a 96-well plate imaged and analyzed using a CellProfiler pipeline for measuring integrated intensity, mean intensity, or nuclear to cytoplasmic ratio of mean intensities [76]. LC3B stained cells were marked as positive for autophagy only if very bright puncta were detected.

### 2.7. Dye Staining Protocols

For the Live/Dead assay, 1 µM Calcein Green AM and 0.5 µM ethidium homodimer-1 (L3224, ThermoFisher) were added to PBS along with 10 µg/mL of Hoechst to make the staining solution. Half the medium was removed from the human neuronal cells that had been incubated with treatments for 3 days and replaced with staining solution. This was conducted four times to make sure that cells did not detach. Cells were then incubated for 15 min at 37 °C and imaged. For resting membrane potential staining, DiBAC4(3) was used. U87 cells were seeded at 10,000 cells per mL on Matrigel-coated plates as described above. Cells were treated with drugs for 6 days with changes in media every other day. On day 6 the media was removed and washed once with dye buffer consisting of Hank’s Balanced Salt Solution (HBSS) with 20 mM HEPES at pH 7.4, and then the staining solution, consisting of dye buffer with 2 µM DiBAC4(3), was added to the cells and allowed to incubate for 30 min at 37 °C. The staining solution was then removed, and fresh staining solution containing the treatments were added to the cells. For cytoplasmic calcium staining, Fluo-4 AM was used. The staining solution consisted of a dye buffer with 4 µM Fluo-4 AM and a 1:1 ratio of Pluronic F-127 (20% in DMSO) and allowed to incubate for 30 min at room temperature. The staining solution was then removed, and fresh dye buffer was added and was allowed to incubate for 20 min at 37 °C. Then the dye buffer containing the treatments were added to the cells. For cytoplasmic pH staining, pHrodo Green was used. The staining solution consisted of a dye buffer with a 1:1000 dilution of the pHrodo Green stock and a 1:100 dilution of the PowerLoad concentrate (P35373, ThermoFisher). The cells were allowed to incubate for 30 min at 37 °C. The staining solution was removed and washed once with dye buffer, then fresh dye buffer containing the treatments were added and allowed to incubate for 5 min at 37 °C. A calibration curve was made by instead adding the components of the intracellular pH calibration kit (P35379, ThermoFisher). For lysosomal pH staining, LysoSensor Green was used. The staining solution consisted of a dye buffer with a 1 µM dilution of LysoSensor Green DND-187 (L7535, ThermoFisher). The cells were incubated as above, and the staining solution was removed and replaced with a dye buffer containing 2.5 µg/mL Hoechst 33342 for 10 min. Hoechst was removed, and cells were washed with dye buffer one time before adding dye buffer containing the treatments. Cells were imaged using an EVOS M7000 system outfitted with a GFP filter cube for stains and a DAPI filter for Hoechst. Images were then analyzed using a CellProfiler pipeline.

### 2.8. Electrophysiology

The SyncroPatch 384PE platform (Nanion Technologies^®^, Munich, Germany) was used to perform the automated high-resolution whole-cell patch-clamp recordings in a 384-well plate format. The experiments were performed within one hour after the harvesting process. The assays were carried out in medium resistance single-hole chips (4–5 MΩ).

Cells were harvested 48–72 h after seeding 1–2 × 10^6^ cells in T175 culture flasks (Falcon), then cells were rinsed with PBS (5 mL) and treated with 3 mL Accutase (STEMCELL Technologies, Vancouver, BC, Canada) for 5 min at 37 °C, re-suspended in 10 mL of serum-free media and pelleted at 160 g for 5 min at RT. The supernatant was discarded, and cells were re-suspended in serum-free DMEM medium with high glucose and no phenol red or sodium pyruvate (31053-028, ThermoFisher), and physiological extracellular solution (pECS) 50% (*v*:*v*). The cells were kept until the moment of the experiment in a temperature-controlled dedicated reservoir at 10 °C and shaken at 200 rpm as described [77].

The chip was primed with the following solutions (in mM), physiological extracellular solution (pECS) 10 HEPES, 140 NaCl, 5 Glucose, 4 KCl, 2 CaCl_2_, 1 MgCl_2_, 295–305 mOsm pH 7.4 (NaOH). Internal recording solution (in mM) 20 EGTA, 50 KCl, 10 NaCl, 60 KF, 10 HEPES at pH 7.2, and 285 mOsm. 15 µL of the cell suspension (50% *v*/*v* pECS/Medium no serum) was added to each well to a final density of 50–80 K cells/mL. Cell capture was promoted by holding negative pressure of −100 mbar for 20 s. After the capture, successive hyperpolarization steps from −30 mV to −100 mV were applied to foster the electrical seal formation. The seal was enhanced by transient addition of a high Ca^2+^ extracellular solution (80 mM NaCl, 3 mM KCl, 10 mM CaCl_2_, 10 mM MgCl_2_, 10 mM HEPES at pH 7.4 and 298 mOsm. High Ca^2+^ solution was washed out by successive external exchanges replacing half of the volume of the well each time with the external recording solution. All recording solutions were prepared with ultrapure MilliQ water (18 MΩ-cm).

After the formation of the Giga-seal, a −250 mbar pressure was applied to break the membrane patch. Once in whole-cell configuration, the resting membrane potential was monitored in current-clamp configuration “I = 0”, the values shown correspond to the average of seven 1 s length sweeps, with an inter-sweep time of 10 s. Cells were also recorded in voltage-clamp mode to characterize the different voltage-dependent conductance present in NG108-15 cells.

Compounds in DMSO stocks were dissolved 1–2 h before the experiment in pECS to a final concentration of 2× the working concentration. 40 µL of the 2× compound solution was applied to each well to a final volume of 80 µL. Before the compound addition, a 5 min recording in 0.2% DMSO was used as a baseline to normalize the changes in the V_mem_.

## 3. Results

### 3.1. Bioelectric Compounds and Combinations with the Proton Pump Inhibitor, Pantoprazole Stop Proliferation of NG108-15 Cells in High Serum and Shift Proportion of Cells in Late S, G2, M

NG108-15 cells containing the FUCCI cell cycle reporter and palmitoyl-mTurquoise2 fluorescent membrane tag were incubated with compounds by themselves and in combination, in media containing high serum (known to be prohibitive for differentiation in this line), to test for their ability to suppress proliferation (Supplementary Figures S1 and S2). The compounds chosen for testing included compounds known to alter the membrane potential of the cell by decreasing proton efflux [78], increasing potassium efflux [79], or decreasing sodium influx [80], and others chosen for their potential combinatorial effects with the ion modulating drugs, including cell cycle-specific disruptors and autophagy-inducing compounds (Supplementary Table S1). In addition, we also tested the most clinically relevant compound currently used in the standard treatment of glioblastoma, temozolomide (TMZ). A subset of the compounds exhibiting the highest efficacy in the initial screens are shown in Table 1.

**Figure 1:**
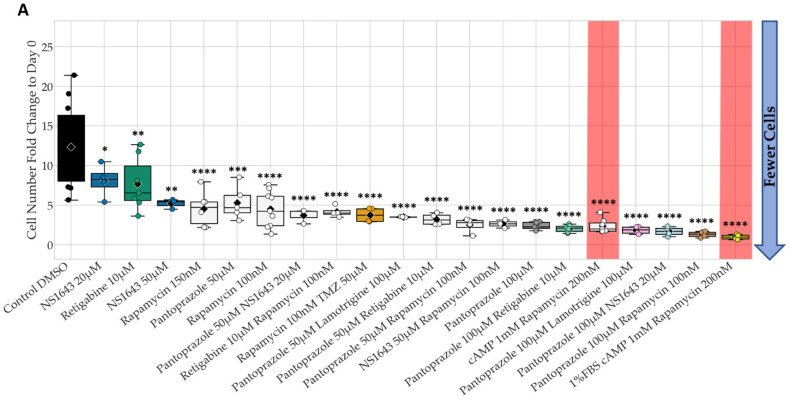

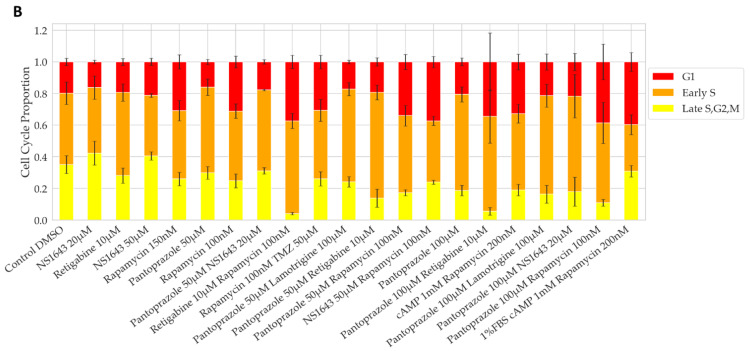
NG108-15 Proliferation is Significantly Lowered with Bioelectric Treatment and Show Changes in Cell Cycle Ratios. (**A**) Fold change to start cell counts (cells at day 6/cells at day 0) after 6 days of treatment. Low values indicate less cell growth. Colors indicate treatments followed up for further analysis, and hues represent concentrations and combinations. Red shaded treatments correspond to positive controls that cannot be used clinically. Only treatments with significant values are shown out of 33 treatments compared to DMSO control. ****: *q* < 0.0001 ***: *q* < 0.001, **: *q* < 0.01, *: *q* < 0.05 (one-way ANOVA with FDR post hoc analysis *n >* 3 biological replicates). (**B**) FUCCI cell cycle data at day 6. Increased red and orange fractions indicate cell cycle arrest at G1 or G1 to S transition.

Many of the tested compounds showed a significant difference in proliferation from the controls after 6 days of treatment, but only a few of these were explored in more detail due to their novelty (Table 1). Figure 1 shows the best of the individual and combination treatments chosen when compared to control at day 6. Note we did not include chlorzoxazone combinations in the NG108-15 analysis shown, due to its poor performance in the U87 cell line. The stacked bar graphs show the ratio of cells in different parts of the cell cycle as indicated by the FUCCI cell cycle reporter.

The proliferation of NG108-15 cells treated with NS1643, a human Ether-a-go-go (hERG) activator and KCNQ2, KCNQ4, KCNQ2/3 potassium channel potentiator [85] at 20 µM and 50 µM alone were able to lower proliferation significantly about 1.6- and 2.4- fold decrease compared to control respectively (Figure 1A). In fact, NS1643 at 20 µM was very effective at lowing cell proliferation when combined with pantoprazole, a proton pump inhibitor known to inhibit the expression of the vacuolar-ATPases (V-ATPases) in human gastric adenocarcinoma [86]; this combination worked significantly better than pantoprazole or NS1643 alone, with a fold decrease of 7.2. NS1643 at 50 µM also significantly lowered proliferation when combined with rapamycin (autophagy inducer), with a 4.6-fold decrease and worked better than rapamycin or NS1643 alone. Retigabine, which opens the voltage-activated potassium channels KCNQ2-5 [87,88], significantly lowered cell proliferation by about 1.6-fold decrease compared to control, but its combination with rapamycin or pantoprazole worked better than any of those compounds alone with a 2.9 and 6-fold decrease as compared to control, respectively. Pantoprazole at 100 µM, however, was the most effective compound alone (5.1-fold decrease) or in combination with lamotrigine which blocks voltage-gated sodium channels [89], or NS1643, or rapamycin, with fold decreases compared to control of 6.8, 7.2, and 9.3 respectively. These three combinations worked better than one of our positive controls, which consisted of a treatment of 1 mM cAMP with rapamycin at 200 nM in full serum media (5.1-fold decrease). This same positive control treatment is known to terminally differentiate these cells when in low serum [90], which we confirmed. However, the use of cAMP is problematic clinically due to its many off-target effects [91]. The cell cycle data in Figure 1B reveal that pantoprazole increases the proportion of cells in early S and that its combinations can also increase the proportion of cells in G1. Rapamycin treatment alone increased the proportion of cells in G1, while NS1643 treatment did not seem to affect the cell cycle proportion. It is worthwhile to note that these compounds were effective on NG108-15 cells, while temozolomide (TMZ), the standard glioblastoma treatment, was not, a situation found in many GBM cases [92].

**Figure 2:**
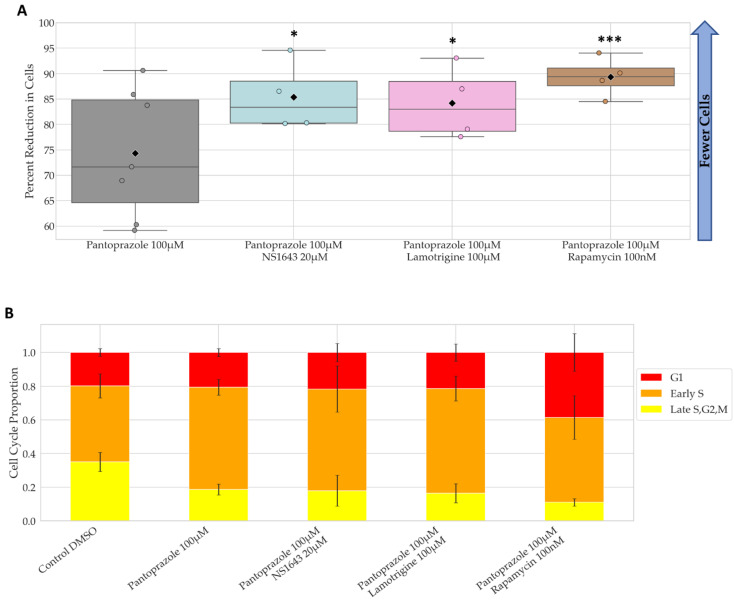
Combinations of Pantoprazole with Bioelectric Compounds Significantly Decrease Proliferation Compared to Pantoprazole Alone and Show Changes in Cell Cycle Ratio. (**A**) Percent reduction in cells compared to control after 6 days of treatment. Treatments that were significantly more effective than pantoprazole alone are shown out of 32 treatments. Statistical analysis was conducted on the log2 of the fold change in cell number to control on day 6. ***: *q* < 0.001, *: *q* < 0.05 (one-way ANOVA with FDR post hoc analysis *n >* 3 biological replicates). (**B**) FUCCI cell cycle data at day 6. Increased red and orange fractions indicate cell cycle arrest at G1 or G1 to S transition.

The combination of pantoprazole with lamotrigine at 100 µM, NS1643 at 20 µM, and rapamycin at 100 nM were the only combinations that showed significantly more efficacy than pantoprazole alone at reducing cell proliferation after 6 days of treatment, with the combination with rapamycin showing the most significant difference (Figure 2A). The reductions in cell number for these combinations compared to control were 85%, 86%, and 90%, respectively. The proportion of cells in G1 and early S only slightly increased for pantoprazole treatments in combination with lamotrigine and NS1643, but the combination with rapamycin did increase the G1 proportion (Figure 2B). Taken together, these data reveal that several FDA approved drugs for human use can be repurposed in combination and can significantly reduce cancer cell proliferation.

### 3.2. Bioelectric Drugs in Combination with Pantoprazole Have a Lasting Effect on NG108-15 Proliferation after Treatment Is Removed

**Figure 3:**
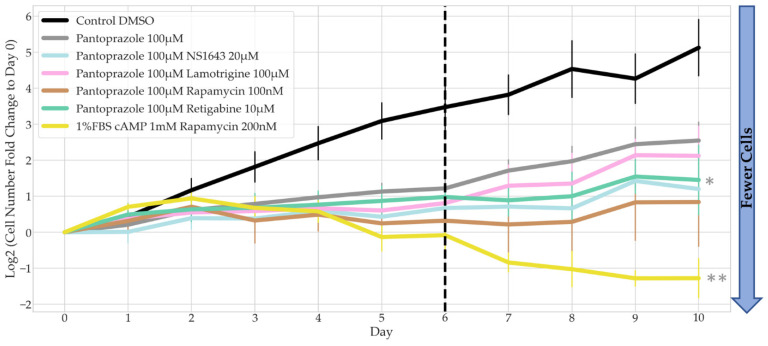
Recovery Test of Bioelectric Treatments in Combination with Pantoprazole in NG108-15 FUCCI Cells. The log2 of the fold change in cell counts to Day 0 were recorded for 10 days. Dotted line marks the day on which drug treatment was removed and replaced with control media (*n >* 3 biological replicates). Combination drug treatment slopes from Day 6 to Day 10 were compared to pantoprazole alone, significance is shown with grey stars next to the corresponding line **: *p* < 0.01, *: *p <* 0.05 (one-way ANOVA with Dunnett post hoc analysis *n >* 3 biological replicates).

To understand whether our treatments have a persistent effect on the cells (stop the proliferation of the cells even after the drugs were withdrawn), we performed a recovery test (Figure 3). Cells were treated with the drugs for 6 days, and then the drugs were removed (demarcated by the dashed line), after which the cells were cultured in media containing no drugs for another 4 days.

Although the three combinations with pantoprazole showed significantly less cell proliferation after 6 days of treatment, the cells showed some recovery after treatment was removed (Figure 3). The slope of each combinatorial treatment from day 6 to day 10 was compared to pantoprazole alone. Pantoprazole in combination with retigabine was the only treatment combination that was significantly different, except the positive control, which showed no recovery, indicative of cells that had terminally differentiated. One thing to note was that the treatment of pantoprazole with retigabine, although not significantly different than pantoprazole at day 6, did show fewer cells than the treatment of pantoprazole with lamotrigine at day 10. Therefore, we decided to perform further analysis on this combination.

### 3.3. Treatments That Were Successful in Reducing the Proliferative Capacity of NG108-15 Cells Were Also Largely Successful in U87 Cells

**Figure 4:**
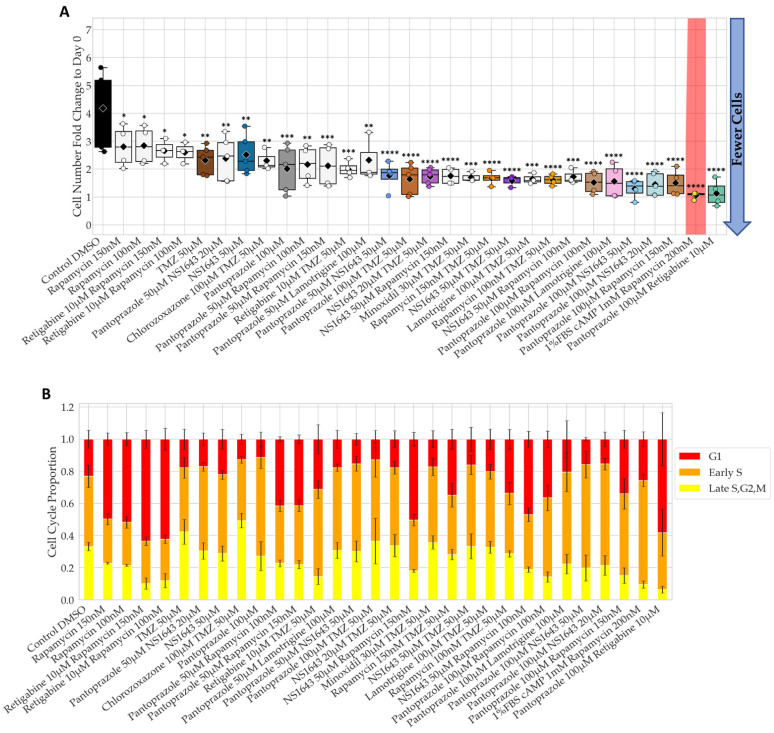
Bioelectric Drugs in Combination with Each Other, Pantoprazole, and TMZ Reduced Proliferation in U87 cells and Changed the Cell Cycle Ratio Compared to Control. (**A**) Fold change to start cell counts (cells at day 6/cells at day 0) after 6 days of treatment. Low values indicate less cell growth. Colors indicate treatments followed up for further analysis and hues represent concentrations and combinations. Red shaded treatment corresponds to positive control that cannot be used clinically. Only treatments with significant values are shown out of 42 treatments compared to DMSO control. ****: *q* < 0.0001 ***: *q* < 0.001, **: *q* < 0.01, *: *q* < 0.05 (one-way ANOVA with FDR post hoc analysis *n >* 3 biological replicates). (**B**) FUCCI cell cycle data at day 6. Increased red and orange fractions indicate cell cycle arrest at G1 or G1 to S transition.

**Figure 5:**
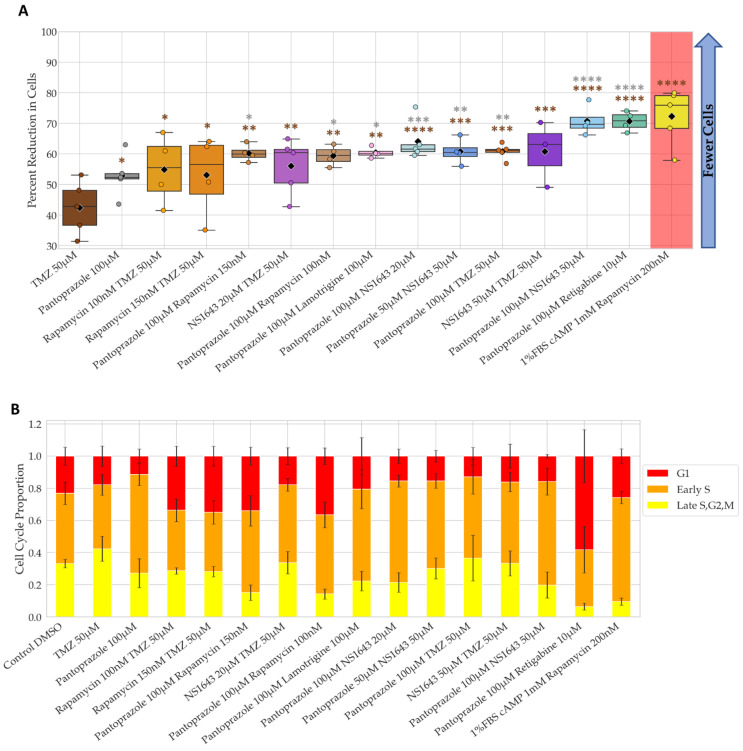
Treatments with Bioelectric Compounds and Pantoprazole or TMZ were Significantly Better than TMZ or Pantoprazole Alone at Reducing Proliferation in U87 cells and Changed the Cell Cycle Ratio Compared to Control. (**A**) Percent reduction in cells compared to control after 6 days of treatment. Treatments that were significantly more effective than TMZ alone are shown out of 42 treatments with brown stars. Treatments with pantoprazole that were significantly better than pantoprazole alone are shown out of 18 treatments with grey stars. Red shaded treatment corresponds to positive control that cannot be used clinically. Statistical analysis was conducted on the log2 of the fold change in cell number to control on day 6. ****: *q* < 0.0001, ***: *q* < 0.001, **: *q* < 0.01, *: *q* < 0.05 (one-way ANOVA with FDR post hoc analysis *n >* 3 biological replicates). (**B**) FUCCI cell cycle data at day 6. Increased red and orange fractions indicate cell cycle arrest at G1 or G1 to S transition.

Many of the significant treatments in NG108-15 cells were also significantly effective in human glioblastoma U87 cells compared to the control after 6 days (Figure 4). Notably, NS1643 at 50 µM significantly decreased cell proliferation as compared to control by 1.7-fold but was much more effective when combined with pantoprazole, rapamycin, or temozolomide (TMZ) with a fold decrease to control of 3.3, 2.7, and 2.5 respectively. Pantoprazole also worked very well in this cell line with a significant percentage reduction in cells to control of 54% and showed very significant differences in cell proliferation as compared to control when combined with rapamycin, retigabine, NS1643, lamotrigine, and TMZ with a percent reduction in cells of 60%, 72%, 72%, 61%, and 61% respectively (Figure 5A). TMZ was very effective at reducing cell number as compared to control in U87 cells (43% decrease), but combinations with rapamycin, pantoprazole, or NS1643 significantly increased the effectiveness up to 55%, 61%, 61% reduction in cells compared to control respectively (Figure 5A). The cell cycle data in Figure 4B shows that some of the most effective combinations increased the G1 and early S proportion of cells but not all. Pantoprazole showed its characteristic increase in the early S proportion of cells and rapamycin, an increase in the G1 proportion as seen in the NG108-15 cells. TMZ and NS1643 treatment did not show a large change in the proportion of cells in each stage of the cell cycle as compared to control. TMZ in combination with rapamycin increased the proportion of cells in G1, and combination with NS1643 increased the proportion of cells in early S as compared to TMZ alone (Figure 5B). Pantoprazole combinations consistently showed a larger proportion of cells in early S as compared to TMZ alone, with a complimentary decrease of cells in late S, G2, and M (Figure 5B).

Unfortunately, we were not able to obtain an accurate proportion of cells in each cell cycle stage for the best treatment in U87 cells, pantoprazole with retigabine, due to an autofluorescent aggregation in the cytoplasm that obscured the FUCCI reporter signal.

The same combinations that were significantly better than pantoprazole in NG108-15 cells were also significant in U87 cells, along with the added combinations of TMZ or retigabine (Figure 5A). Pantoprazole in combination with rapamycin, lamotrigine, NS1643, TMZ or retigabine were all significantly better than pantoprazole alone. The most significant combinations were with NS1643 or retigabine, a 1.6-fold decrease than pantoprazole alone. The combination of pantoprazole with NS1643 was so effective that cutting the pantoprazole concentration by half and combining it with NS1643 at 50 µM was significantly more effective than pantoprazole at 100 µM alone (1.2-fold decrease). The characteristic increase in G1 when pantoprazole was used in NG108-15 cells was also seen in U87 cells. Additionally, when pantoprazole was combined with rapamycin or NS1643 an increase in the proportion of the cells in early S was seen, with a complementary decrease in late S, G2, and M (Figure 5B). Thus, we conclude that NS1643, retigabine, rapamycin, lamotrigine, and pantoprazole are also effective in a human glioblastoma cell line, and NS1643 or pantoprazole potentiate the action of the standard TMZ treatment.

### 3.4. Bioelectric Drug Combinations including Pantoprazole Reduce U87 Proliferation after Treatment Is Removed

**Figure 6:**
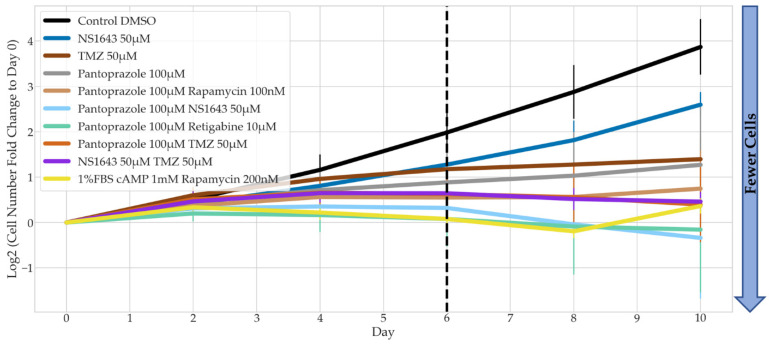
Recovery Test of Bioelectric Treatments in Combination with Pantoprazole or TMZ in U87 cells. The log2 of the fold change in cell counts to Day 0 were recorded for 10 days. Dotted line marks the day on which drug treatment was removed and replaced with control media (*n* > 3 biological replicates). Combination drug treatment slopes from Day 6 to Day 10 were compared to pantoprazole or TMZ alone, but no significance was found (one-way ANOVA with Dunnett post hoc analysis *n* > 3 biological replicates). 3.5. Electrophysiology of NG108-15 Cells Show Changes in Resting Membrane Potential Induced by Treatment.

The combination of pantoprazole with rapamycin shows a similar recovery slope after day 6 as pantoprazole alone (Figure 6). However, combinations of pantoprazole with retigabine or NS1643 at 50 µM show reduced recoveries compared to pantoprazole alone, although not significantly so. TMZ treatment alone only showed a slight increase in proliferation after treatment was removed, and NS1643 alone showed a high increase in proliferation. However, the combination of NS1643 with TMZ did not show an increase in proliferation after removal of treatment, but the recovery slope was not significantly different from TMZ alone. Surprisingly the positive control using cAMP in combination with rapamycin started proliferating after day 8, revealing that the bioelectric drugs have a more stable effect on the cells than even the powerful cAMP signal.

We performed electrophysiological recordings to determine the change in resting membrane potential of NG108-15 cells treated with the compounds that significantly reduced cell proliferation as compared to control immediately after application. We measured the baseline V_mem_ first and recorded the membrane potential continuously for 3 min upon compound treatment. We then calculated the difference in V_mem_ post treatment.

**Figure 7:**
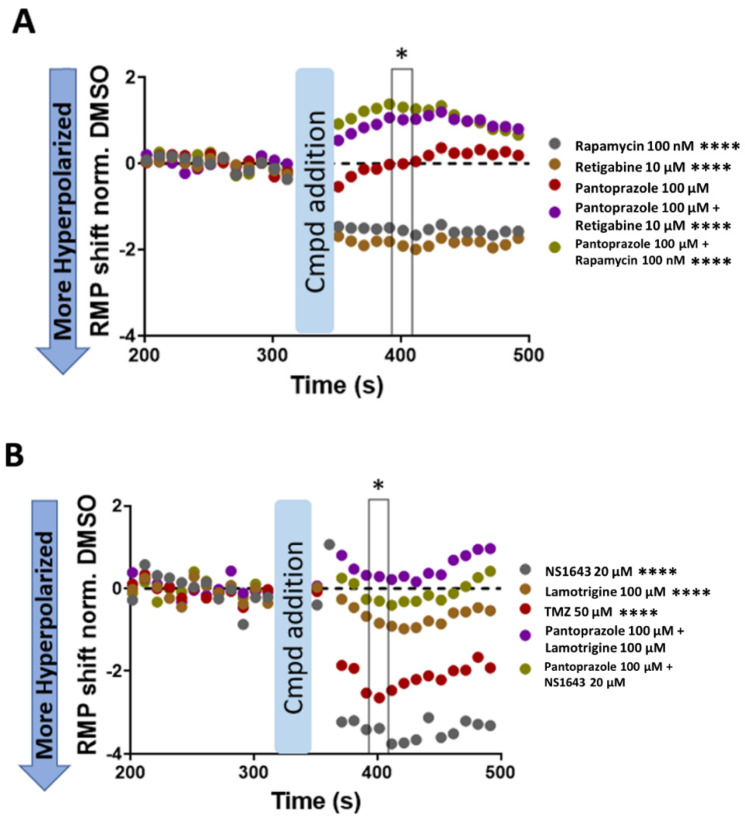
Resting Membrane Potential Changes Caused by Treatments in NG108-15 FUCCI Cells. The change in resting membrane potential is normalized to DMSO control. The negative values represent an increase in hyperpolarization. (**A**,**B**) were conducted on different days. There were *n* = 55–63 cells per condition. Statistics were calculated for the 400 s time point (black box with star on top). Significant values shown next to legend. ****: *p <* 0.0001, were calculated using an ANOVA with Dunnett post hoc analysis.

Rapamycin, retigabine, NS1643, TMZ, and lamotrigine all significantly hyperpolarized the cells as compared to the control (Figure 7). Retigabine, lamotrigine, and NS1643 are known hyperpolarizing agents, but TMZ has been shown to depolarize U373 glioma cells [93]—an effect opposite to what we observed in NG108-15 cells, possibly due to differential expression levels of KCa3.1 channels in these two cell lines (not confirmed). Rapamycin also hyperpolarized the NG108-15 cells, a novel effect suggesting that existing cancer drugs could have bioelectric mechanisms of action that are not yet recognized. Surprisingly, while pantoprazole did not have an immediate effect on the membrane potential of the cells, its combination with retigabine and rapamycin, which both individually hyperpolarize the cells, instead depolarized the cells. The combination of pantoprazole with lamotrigine and NS1643 did not observably change the membrane potential as compared to control.

### 3.5. NG108-15 Cells Express Neuronal Markers with Drug Treatments after 6 Days

**Figure 8:**
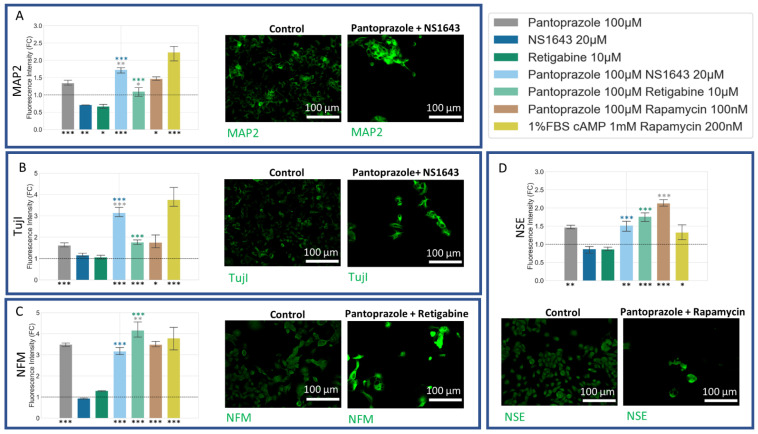
Differentiation Analysis of NG108-15 Cells Reveals that Treatments with Pantoprazole Increased Neuronal Markers after 6 days. Immunofluorescence of cells was analyzed with CellProfiler and quantified for integrated fluorescence intensity. (**A**) Stain of Microtubule Associated Protein 2 (MAP2). (**B**) Stain of Neuron-Specific Class III β-Tubulin (Tuj I). (**C**) Stain of Neural Filament Medium Chain (NFM). (**D**) Stain of Neuron-Specific Enolase (NSE). Treatments corresponding to the colored bars are outlined in the figure itself. The log of the fold change in intensity was compared between single treatments and combined treatments, except the positive control, with significant values shown in the color of the treatment compared. The initial fluorescence intensities were compared to their corresponding control, with significant values shown under the bars as, ***: *p <* 0.001, **: *p* < 0.01, *: *p <* 0.05 (one-way ANOVA with Tukey post hoc analysis *n >* 3 technical replicates).

We next wanted to ask whether, in addition to the effects on proliferation, our treatments also exerted a differentiating influence, which could be beneficial with respect to the future behavior of treated cells in vivo. Differentiation markers for neuronal lineage were used to stain NG108-15 cells incubated for six days with the most effective treatments observed in the proliferation data (Figure 8). We found that treatment with pantoprazole at 100 µM combined with NS1643 at 50 µM or with rapamycin at 100 nM consistently showed a significant increase in neuronal differentiation markers, including Microtubule Associated Protein 2 (MAP2) [94], Neuron-Specific Class III β-Tubulin (TujI) [95], Neuron-Specific Enolase (NSE) [96], and Neural Filament Medium Chain (NFM) [97]. The combination of pantoprazole with retigabine at 10 µM showed increases in all but MAP2. Combinations of pantoprazole with either NS1643 or retigabine showed a significant increase in neuronal markers compared to either drug alone. The combination of NS1643 with pantoprazole showed higher levels of both MAP2 and Tuj1, than for pantoprazole alone. Retigabine in combination with pantoprazole showed higher immunoreactivity of NFM as compared to pantoprazole alone. These observations suggest that these treatments are pushing the treated cells toward a more differentiated state.

### 3.6. NG108-15 Cells Express Astrocyte and Other Differentiation Markers after Drug Treatments for 6 Days

**Figure 9:**
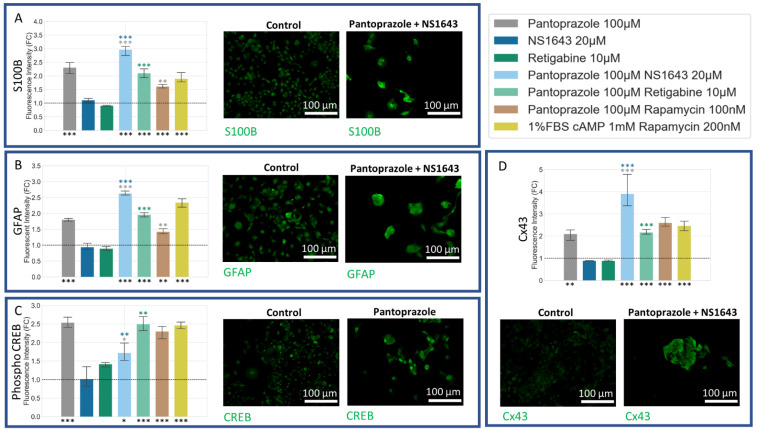
Differentiation Analysis of NG108-15 Cells Reveals that Treatments with Pantoprazole Increased Astrocytic and Differentiation Markers after 6 days. Immunofluorescence of cells was conducted and analyzed with CellProfiler and measured for integrated fluorescence intensity. (**A**) Stain of S100 calcium-binding protein B (S100B). (**B**) Stain Glial Fibrillary Acidic Protein (GFAP). (**C**) Stain of the phosphorylated cAMP-Response Element Binding Protein (Phospho CREB). (**D**) Stain of Connexin 43 (Cx43). Treatments corresponding to the colored bars are outlined in the figure itself. The log of the fold change in intensity was compared between single treatments and combined treatments, except the positive control, with significant values shown in the color of the treatment compared. The initial fluorescence intensities were compared to their corresponding control, with significant values shown under the bars as, ***: *p <* 0.001, **: *p* < 0.01, *: *p <* 0.05 (one-way ANOVA with Tukey post hoc analysis *n >* 3 technical replicates).

Staining cells treated for 6 days with S100 calcium-binding protein B (S100B) [98] and Glial Fibrillary Acidic Protein (GFAP) [99] markers revealed differentiation of NG108-15 cells towards an astrocytic/oligodendrocytic or astrocytic lineage, respectively (Figure 9A,B). Both markers were significantly upregulated in all combination treatment groups as well as in pantoprazole alone. The combination of NS1643 with pantoprazole showed significantly higher S100B and GFAP immunoreactivity than with pantoprazole alone. All treatments but NS1643 alone showed a significant increase in CREB, known to play an important role in driving differentiation [16,100,101,102,103] (Figure 9C). Pantoprazole alone, along with its combinations, also showed an increase in connexin 43 (Cx43) expression, a known marker for glioblastoma differentiation [104].

### 3.7. NG108-15 Cells Show an Increase in p27^Kip1^ and Senescence Markers When Treated with Pantoprazole Alone or in Combination with Hyperpolarizing Compounds

**Figure 10:**
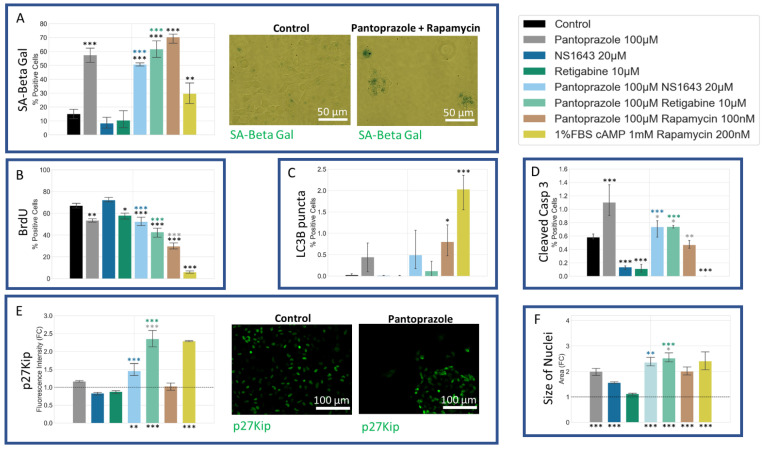
Senescence and Proliferation Analysis of NG108-15 Cells Reveals that Treatments with Pantoprazole Increased Senescence, Decreased BrdU Incorporation, and Increased a p27^Kip1^ after 6 days. A senescence associated beta-galactosidase stain was conducted and scored by eye. Immunofluorescence of cells was conducted and analyzed with CellProfiler for integrated fluorescence intensity or presence or absence of a cellular signal. (**A**) Stain of senescence associated beta-galactosidase stain (SA-Beta Gal). (**B**) Stain of bromodeoxyuridine incorporation (BrdU). (**C**) Stain of the microtubule-associated protein light chain 3 B (LC3B). (**D**) Stain of cleaved caspase 3 (Casp 3). (**E**) Stain of cyclin-dependent kinase inhibitor 1B (p27Kip). (**F**) Size of Nuclei, determined by area of the Hoechst stain. Treatments corresponding to the colored bars are outlined in the figure itself. The log of the fold change in intensity was compared between single treatments and combined treatments, except the positive control, with significant values shown in the color of the treatment compared. The initial fluorescence intensities were compared to their corresponding control, with significant values shown under the bars. The logit of the percent positive cells was compared between single treatments and control, in cases of 0 values, the arcsine transformation was used. Significance was expressed as, ***: *p <* 0.001, **: *p* < 0.01, *: *p <* 0.05 (one-way ANOVA with Tukey post hoc analysis *n >* 3 technical replicates).

We also sought to determine whether our treatments may induce senescence, as this would be an important outcome with respect to the course of the malignancy in vivo. Treatments of NG108-15 cells with pantoprazole alone or in combination with NS1643, retigabine, and rapamycin all showed a very significant increase in a senescence-associated beta-galactosidase activity stain [105] (Figure 10A). To explain this increase, we also looked at the p27^Kip1^ level, which is known to inhibit the cell cycle and cause senescence [106,107,108,109,110,111,112,113] (Figure 10E). Immunoreactivity levels of p27^Kip1^ in all combined treatments except for pantoprazole with rapamycin were elevated. To confirm the senescence phenotype, we also looked at the size of the nuclei, which were significantly larger in the pantoprazole, NS1643, and pantoprazole in combination with NS1643, retigabine, or rapamycin-treated groups (Figure 10F). We also tested the cells for cleaved caspase 3 (Figure 10D), a marker of apoptosis and LC3B (Figure 10C), a marker of autophagy. We found that levels for both these markers were under 1.4% and 2.5%, respectively, after 6 days of treatment which may indicate a small percentage of cells that did not senesce due to the highly heterogeneous nature of these cancer cells. Although low, there was a significant increase in cleaved caspase 3 staining for cells treated with pantoprazole or pantoprazole with NS1643, retigabine, or rapamycin. Surprisingly, we observed that treatment with retigabine or NS1643 decreased the cleaved caspase 3 positive cells significantly as compared to the control. In addition, the LC3B stain showed an increase in cells treated with pantoprazole and pantoprazole with NS1643, although not significant with this assay. However, there was a significant increase in bright LC3B puncta in cells that were treated with any combination containing rapamycin, as was expected (Figure 10C). Proliferation in NG108-15 cells as measured by BrdU incorporation showed a significant decrease with treatments of pantoprazole alone, retigabine alone, and combinations of pantoprazole with NS1643, retigabine, and rapamycin after 6 days (Figure 10B), agreeing with the live cell counts obtained in Figure 1A. Thus, we conclude that the combinatorial treatments that resulted in the lowest cellular proliferation also showed increased markers for senescence, some increase in autophagy, and did not show a large fraction of apoptotic cells.

### 3.8. U87 Cells Show Increases in Neuronal Markers When Treated with Combination Treatments of Hyperpolarizing Drugs and Pantoprazole or NS1643 and TMZ

**Figure 11:**
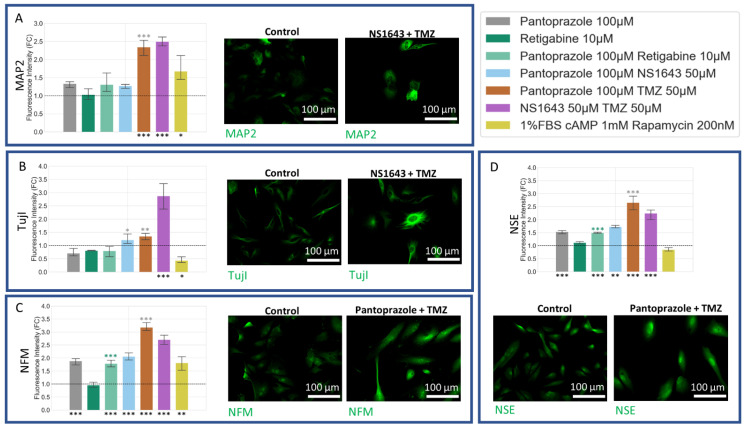
Differentiation Analysis of U87 Cells Reveals that Treatments with Pantoprazole Increased Neuronal Markers after 6 days. Immunofluorescence of cells was conducted and analyzed with CellProfiler for integrated fluorescence intensity. (**A**) Stain of Microtubule Associated Protein 2 (MAP2). (**B**) Stain of Neuron-Specific Class III β-Tubulin (TujI). (**C**) Stain of Neural Filament Medium Chain (NFM). (**D**) Stain of Neuron-Specific Enolase (NSE). Treatments corresponding to the colored bars are outlined in the figure itself. The log of the fold change in intensity was compared between single treatments and combined treatments, except the positive control, with significant values shown in the color of the treatment compared. The initial fluorescence intensities were compared to their corresponding control, with significant values shown under the bars as, ***: *p <* 0.001, **: *p* < 0.01, *: *p <* 0.05 (one-way ANOVA with Tukey post hoc analysis *n >* 3 technical replicates).

U87 cells showed a significant increase in neuronal markers for the combination treatments with TMZ as compared to control (Figure 11). NFM and NSE, similar to what was seen in NG108-15 cells, were also increased in cells treated with pantoprazole or combination treatments with pantoprazole and NS1643 or retigabine (Figure 11C). TujI was only highly elevated in the U87 cells treated with NS1643 and TMZ (Figure 11B). MAP2 showed high levels of immunoreactivity when cells were treated with combinations of TMZ and NS1643 or pantoprazole (Figure 11A). It should be noted that cell staining was highly heterogeneous, with some cells staining more brightly than others with the same treatment.

### 3.9. U87 Cells Also Show Increases in Astrocytic Markers When Treated with Combination Treatments of Hyperpolarizing Drugs and Pantoprazole or NS1643 and TMZ

**Figure 12:**
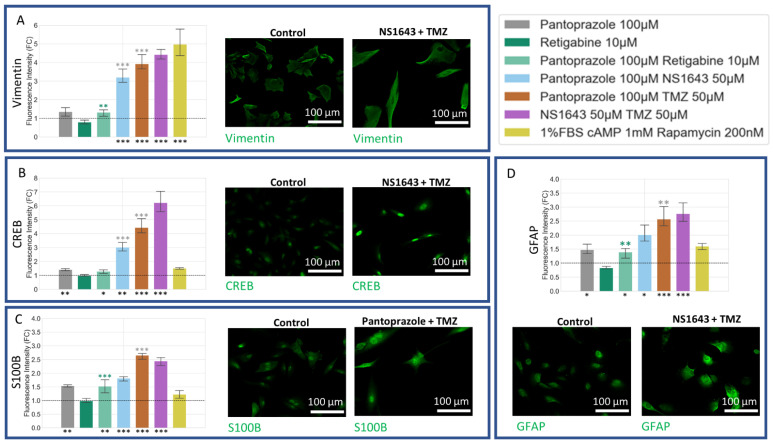
Differentiation Analysis of U87 Cells Reveals that Treatments with Pantoprazole Increased Astrocytic and Differentiation Markers after 6 days. Immunofluorescence of cells was conducted and analyzed with CellProfiler for integrated fluorescence intensity. (**A**) Stain of Vimentin. (**B**) Stain of the phosphorylated cAMP-Response Element Binding Protein (CREB). (**C**) Stain of S100 calcium binding protein B (S100B). (**D**) Stain Glial Fibrillary Acidic Protein (GFAP). Treatments corresponding to the colored bars are outlined in the figure itself. The log of the fold change in intensity was compared between single treatments and combined treatments, except the positive control, with significant values shown in the color of the treatment compared. The initial fluorescence intensities were compared to their corresponding control, with significant values shown under the bars as, ***: *p <* 0.001, **: *p* < 0.01, *: *p <* 0.05 (one-way ANOVA with Tukey post hoc analysis *n >* 3 technical replicates).

Astrocytic differentiation markers were increased in treatments with pantoprazole and pantoprazole in combination with NS1643, retigabine, rapamycin, and TMZ, as well as NS1643 in combination with TMZ (Figure 12). Vimentin, a known marker for astrocytes [114,115], was significantly increased when cells were treated with pantoprazole in combination with NS1643, TMZ, and rapamycin and with NS1643 in combination with TMZ (Figure 12A). CREB (Figure 12B), a known marker for differentiation and S100B (Figure 12C) and GFAP (Figure 12D), markers for astrocytic differentiation, also were increased by these same treatments (Figure 12B). These results agreed well with what was found in NG108-15 cells.

### 3.10. U87 Cells Show Increase in Oligodendrocyte Markers When Treated with Pantoprazole in Combination with Hyperpolarizing Compounds or NS1643 and TMZ

**Figure 13:**
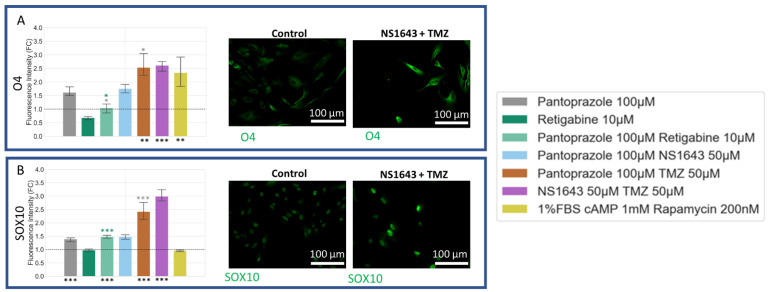
Differentiation Analysis of U87 Cells Reveals that Treatments with Pantoprazole Increased Oligodendrocyte Markers after 6 days. Immunofluorescence of cells was conducted and analyzed with CellProfiler for integrated fluorescence intensity. (**A**) Stain of oligodendrocyte marker O4. (**B**) Stain of the Sry-related HMg-Box gene 10 (SOX10). Treatments corresponding to the colored bars are outlined in the figure itself. The log of the fold change in intensity was compared between single treatments and combined treatments, except the positive control, with significant values shown in the color of the treatment compared. The initial fluorescence intensities were compared to their corresponding control, with significant values shown under the bars as, ***: *p <* 0.001, **: *p* < 0.01, *: *p <* 0.05 (one-way ANOVA with Tukey post hoc analysis *n >* 3 technical replicates).

U87 cells showed increases in O4 (Figure 13A) for all treatments except those containing retigabine. Sox10 levels (Figure 13B) also increased for all treatments, excluding retigabine alone. When taken together, the above data suggest that the differentiating activity of these treatments are not specific to one species or one type of cell line.

### 3.11. U87 Cells Also Show an Increase in p27^Kip1^ and Senescence Markers When Treated with Pantoprazole Alone or in Combination with Hyperpolarizing Compounds

**Figure 14:**
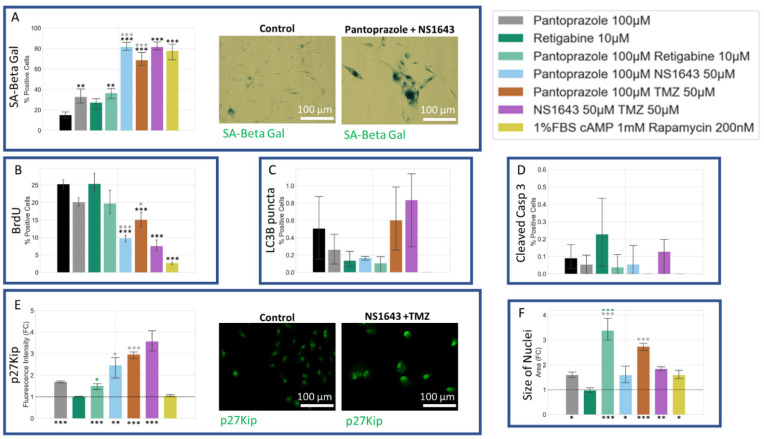
Senescence and Proliferation Analysis of U87 Cells Reveals that Treatments with Pantoprazole or NS164 with TMZ Increased Senescence, Decreased BrdU Incorporation, and Increased a p27^Kip1^ after 6 days A senescence associated beta-galactosidase stain was conducted and scored by eye. Immunofluorescence of cells was conducted and analyzed with CellProfiler for integrated fluorescence intensity or presence or absence of a cellular signal. (**A**) Stain of senescence associated beta-galactosidase stain (SA-Beta Gal). (**B**) Stain of bromodeoxyuridine incorporation (BrdU). (**C**) Stain of the microtubule-associated protein light chain 3 B (LC3B). (**D**) Stain of cleaved caspase 3 (Casp 3). (**E**) Stain of cyclin-dependent kinase inhibitor 1B (p27Kip). (**F**) Size of Nuclei, determined by area of the Hoechst stain. Treatments corresponding to the colored bars are outlined in the figure itself. The log of the fold change in intensity was compared between single treatments and combined treatments, except the positive control, with significant values shown in the color of the treatment compared. The initial fluorescence intensities were compared to their corresponding control, with significant values shown under the bars. The logit of the percent positive cells was compared between single treatments and control, in cases of 0 values, the arcsine transformation was used. Significance was expressed as, ***: *p <* 0.001, **: *p* < 0.01, *: *p <* 0.05 (one-way ANOVA with Tukey post hoc analysis *n >* 3 technical replicates).

We tested U87 cells for the same proliferation, senescence, autophagy, and apoptosis markers that we had tested in NG108-15 cells and found similar results (Figure 14). All treatments showed an increase in senescence markers, with the most significant being the combination of pantoprazole with NS1643, TMZ, and the combination of NS1643 and TMZ (Figure 14A). The size of the nuclei for the combination treatments with the highest levels of senescent cells was also significantly larger than the control, confirming this phenotype (Figure 14F). We also looked at p27^Kip1^ levels and saw similar changes to those observed in NG108-15 cells (Figure 10E). All the treatments with pantoprazole showed an increase in p27^Kip1^ levels, and NS1643 in combination with TMZ showed the highest increase. Cleaved caspase 3 (Figure 14E) and LC3B puncta (Figure 14C) positive cells indicated very low non-significant levels of expression, indicating that the cells were not undergoing a high level of apoptosis or autophagy relative to control, respectively. BrdU incorporation decreased in all treatments that showed lower proliferation (Figure 14B). These data show that the low proliferation of the U87 cells treated with the most successful drug combinations was driven by increased levels of senescence.

### 3.12. U87 Cells Are Hyperpolarized by Treatment with NS1643 and Its Combination with Pantoprazole and the Combination of NS1643 with Pantoprazole or TMZ Increases the Translocation of YAP to the Cytoplasm

**Figure 15:**
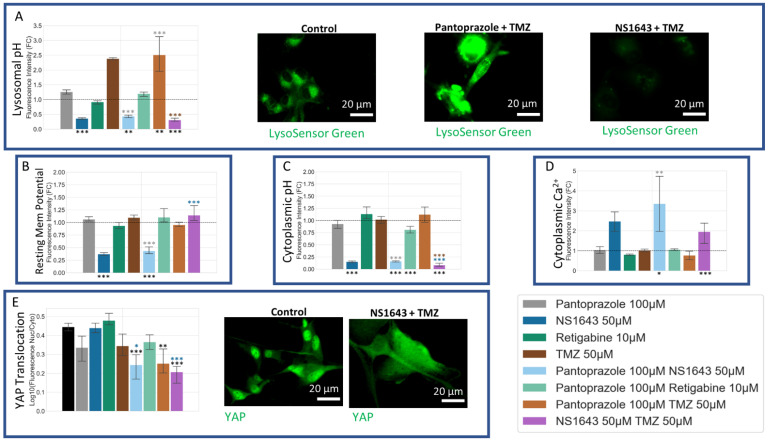
Voltage Dyes Showed that U87 Cells Treated with NS1643 and a Combination of NS1643 and Pantoprazole for 6 Days Showed a Hyperpolarization and YAP Increases its Translocation to the Cytoplasm in NS1643 or Pantoprazole with TMZ, and Pantoprazole with NS1643 Treatment. Immunofluorescence of cells was conducted and analyzed with CellProfiler for integrated fluorescence intensity. Dye assays were analyzed for mean intensity, except for LysoSensor Green which was analyzed for integrated intensity. (**A**) Stain of lysosomal pH with LysoSensor Green, low levels indicate alkalization. (**B**) Dye indicator of membrane voltage, DiBAC4(3), low levels indicate hyperpolarization. (**C**) Dye indicator of cytoplasmic pH, pHRodo Green, low levels indicate alkalization. (**D**) Dye indicator of cytoplasmic calcium, Fluo-4AM, high levels indicate an increase in calcium. (**E**) The ratio of nuclear to cytoplasmic Yes-associated protein (YAP), lower levels indicate translocation to the cytoplasm. Treatments corresponding to the colored bars are outlined in the figure itself. The log of the fold change in intensity was compared between single treatments and combined treatments, except the positive control, with significant values shown in the color of the treatment compared. The initial fluorescence intensities were compared to their corresponding control, with significant values shown under the bars as, ***: *p <* 0.001, **: *p* < 0.01, *: *p <* 0.05 (one-way ANOVA with Tukey post hoc analysis *n >* 3 technical replicates).

We used the voltage dye DiBAC4(3) [116,117] to measure the resting membrane potential of six-day-treated U87 cells (Figure 15B). We found that NS1643 alone and in combination with pantoprazole showed a significant hyperpolarization as compared to control. To investigate further, we used pHRodo Green to see if the internal pH of the cells was being changed in response to treatment (Figure 15C). We saw a dramatic increase in pH with all the treatments that incorporated NS1643, and we saw a slight increase in pH for pantoprazole with retigabine. In addition to the cytoplasmic pH, we wanted to test the lysosomal pH due to reports that pantoprazole de-acidifies the lumen of the lysosome [118,119]. The dye, LysoSensor Green (Figure 15A), showed a dramatic alkalization of the lysosomes when treated by NS1643 alone or in combination. Pantoprazole did not show an alkalinization of the lysosome in the U87 cells, in agreement with what has previously been reported when pantoprazole is delivered in neutral cell culture media at pH 7.4 [120]. In fact, pantoprazole and its combination with TMZ showed a significant increase in lysosomes, which also agrees with that same study performed in neutral pH media [120]. In addition to the dyes, we also tested the ratio of cytoplasmic to nuclear YAP (Figure 15E). Pantoprazole has been found to decrease YAP activity in ovarian cancer and in the liver [121,122]. Since YAP has been found to be a master regulator of the cell cycle, especially in cancer, we were interested to see the effect our treatments would have on this protein [122,123,124,125]. We found that pantoprazole alone did not have a significant effect on the cytoplasmic to nuclear ratio of YAP, but when combined with NS1643 or TMZ, it showed a significant decrease indicative of less YAP in the nucleus as compared to the cytoplasm. This significant decrease in the YAP nucleus to cytoplasmic ratio was also evident for the NS1643 in combination with TMZ treatment. When taken together, these data indicate that NS1643, in combination or alone, can increase cytoplasmic calcium levels, increase cytoplasmic pH, and increase lysosomal pH, but a significant decrease in the YAP nuclear to cytoplasmic ratio is only seen when it is in combination with pantoprazole or TMZ.

### 3.13. Neuronal Cell Toxicity Was Minimal after a Three-Day Treatment with the Top Performing Drugs and Drug Combinations

To determine whether the effects we observed were specific for cancer cells and could be expected to be usable in vivo without harming native neurons, we tested these compounds on human induced pluripotent stem cells derived from fibroblasts and made to commit to a neuronal stem cell lineage. These hiNSCs were differentiated for 7 days in neuronal media and then treated with electroceuticals for 3 days. The short treatment time was necessary to be able to perform a Live/Dead assay without too much cell detachment.

**Figure 16:**
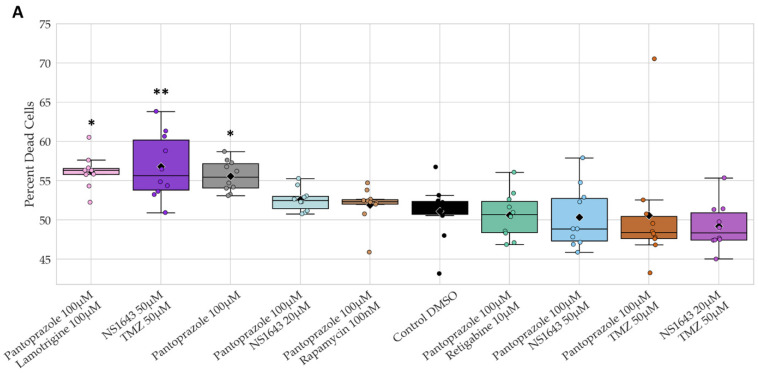

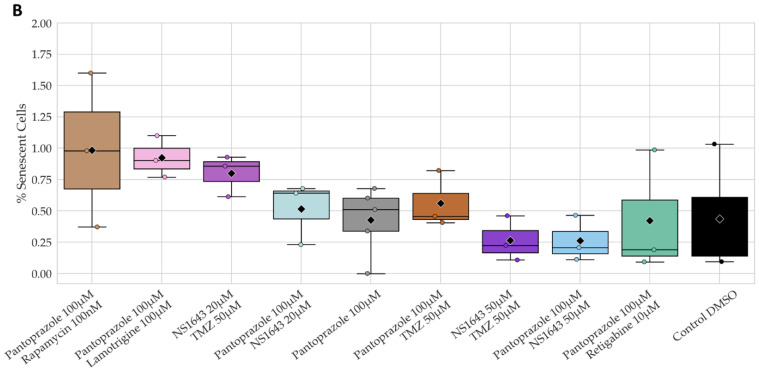
Live/Dead assay and Senescence assay of Human Neuronal Cells After 3 Day Treatment Shows Low Level of Toxicity. Low values indicate less death or senescent cells. (**A**) Live/Dead assay conducted on human neuronal cells cultured with drug for 3 days. (**B**) Senescence assay results of senescence associated beta-galactosidase staining on human neuronal cells cultured with drug for 3 days. Treatments with best reduction of proliferation in NG108-15 or U87 cells are shown out of a 24-sample toxicity screen, with significant values shown. **: *q* < 0.01, *: *q* < 0.05 (one-way ANOVA with FDR post hoc analysis *n >* 3 technical replicates). Increase in percent dead or senescent cells indicative of toxic treatment.

Toxicity analysis by Live/Dead stain in human neurons shows that only three out of 24 treatments showed significant toxicity (Figure 16A). Pantoprazole showed a slight increase in toxicity that was significant compared to control, and pantoprazole with lamotrigine also showed a slight increase but was more significant than pantoprazole alone when compared to the control. NS1643 at 50 µM in combination with TMZ showed the most toxicity when compared to control. However, the difference between the control toxicity and the most toxic combination of NS1643 at 50 µM and TMZ was still only 5.7% higher than control. A senescence assay was also performed on the neurons under all treatments tested in the Live/Dead stain, but no significant differences were found, and all senescence levels were all under 1.75%, a stark contrast to the greater than 50% senescence levels in NG108-15 and U87 cells after 6 days of treatment (Figure 10A and Figure 14A).

## 4. Discussion

### 4.1. Putative Electroceuticals for Cancer

Our drugs were selected based on their predicted effects on V_mem_, which has been shown in amphibian models in vivo to prevent and reverse tumorigenesis and metastatic behavior [34,47]. Indeed, a number of drugs with bioelectric targets, such as ivermectin (a chloride channel drug) [126,127,128,129], salinomycin and monensin (ionophores) [126,130], a variety of potassium channel drugs [25,55,131,132,133,134], and drugs targeting proton pumps [135] have been discovered to have anti-cancer activity in various screens [26,136,137,138,139]. Thus, our combinations of compounds represent novel entries to the field of electroceuticals: the repurposing of known ion channel-targeting drugs to manipulate complex cell outcomes [140,141]. This approach has already been used for the design of interventions to repair birth defects of the brain [142,143]. It is likely that a better understanding of the control of cell behavior, alone and in tissues, will enable much more precisely targeted electroceutical interventions in cancer as part of the goal of normalizing cells as an alternative to traditional chemotherapy [144].

### 4.2. Effects on Proliferation

The NG108-15 hybrid cell line used in this study shows cancer stem cell characteristics, can be easily transfected and selected and has been used to study neuronal differentiation for many years. To find treatments that would be robust, we screened all our compounds in high serum media, which is usually prohibitive for NG108-15 differentiation [90,145,146]. In addition, the high serum media provided an abundance of growth factors that have been shown to be secreted in the peripheral zone of resected GBM tumors and are thought to drive the migration and proliferation of GBM stem cells in the area [10]. The best performing novel combinations were pantoprazole with retigabine, lamotrigine, NS1643, or rapamycin and reduced proliferation when compared to the control by 80%, 85%, 86%, and 90%, respectively. FUCCI analysis showed that the cell cycle proportion was dominated by cells in either G1 or early S. All these compounds are already FDA approved for other conditions except for NS1643.

We then used the best performing novel combinations to inform a screen of compounds on a human glioblastoma cell line, U87 (ATCC). Our proliferation assays showed 13 drug or drug combinations that reduced proliferation compared to control more significantly than the leading glioblastoma treatment, TMZ. Of these 13, the top four treatments were combinations of pantoprazole or NS1643 with TMZ, and the combination of pantoprazole with retigabine or NS1643, which reduced proliferation when compared to control by 62%, 63%, 71%, and 74%, respectively. FUCCI analysis showed that the cell cycle proportion was again dominated by cells in either G1 or early S.

### 4.3. Effects on Differentiation

Differentiation therapy for GBM is an alternative treatment strategy that could possibly overcome the issue of reoccurrence after resection of the tumor [15]. For differentiation therapy to be successful, treatment needs to be effective at clinically relevant concentrations, and differentiation needs to be permanent, without cell cycle re-entry after treatment is stopped. This study showed that hyperpolarizing compounds in combination with pantoprazole can be used to arrest the cell cycle of proliferating cells and drive them towards partial differentiation and senescence.

NG108-15 cells treated with the most successful drug combinations showed neuronal, astrocytic, and oligodendrocyte differentiation markers as well as senescence markers. The mixed nature of differentiation markers in NG108-15 cells has been reported previously [147] and might be due to their hybrid neuroblastoma/glioma status. Many of the combinations of drugs we tested in NG108-15 cells were also successful in U87 cells and resulted in dramatically less proliferation, less progression through the cell cycle and a significant increase in differentiation markers, which need the cell cycle to be arrested long enough to accumulate [148]. The mixed nature of the differentiation markers that were elevated in U87 cells shows that this line can differentiate into the neuron, astrocyte, and oligodendrocyte lineages, in agreement with past studies [149,150]. It is important to note that many of the differentiation markers were heterogeneously expressed and that there is a possibility that in each sample, there exist a multitude of differentiated phenotypes instead of just one type. Connexin 43 was also upregulated in treated NG108-15 cells, showing that this marker of cell-to-cell communication was increased as cells went to a more differentiated state, as supported by a previous study [104]. Interestingly, both NS1643 and retigabine are known to work on KCNQ2/3 channels, which are important for neuronal differentiation [151], and NS1643 activates hERG channels which have also been shown to be important in neuronal differentiation [65].

### 4.4. Electrophysiology, pH, and Cell Cycle Regulation

NS1643, a hERG channel opener and potassium modulator which also potentiates KCNQ2, KCNQ4, and KCNQ2/3 [85,152], was shown to hyperpolarize both cell lines that we tested and was one of the most successful single treatments. In combination with pantoprazole, it could arrest the NG108-15 cell cycle long enough to enable differentiation in a high serum medium and work well in U87 cells. Treatment with NS1643 in combination with pantoprazole showed a significant effect that was not seen with either treatment alone. Assays in U87 cells showed that this novel combinatorial treatment caused the pH of the cytoplasm and lysosomes to alkalize drastically and that cytoplasmic calcium increased significantly along with an increase in the cell cycle inhibitor p27^Kip1^ and an increase in senescence-associated beta-galactosidase positive cells. In addition, combination treatments with NS1643 also resulted in a decrease in YAP nuclear to cytoplasmic ratio, elevated levels of which are known to accelerate cancer cell cycle progression, proliferation, therapy resistance, and metastasis [124,125,153]. Thus, both YAP translocation and increased p27^Kip1^ levels were found in the cells that showed the most senescence, confirming what other studies have reported: that both proteins are involved in senescence [111,154]. Proliferation under these senescence-inducing treatments was reduced dramatically, and U87 cells did not re-enter the cell cycle up to 4 days after treatment was removed. The combination of NS1643 with TMZ was also very effective at reducing proliferation in U87 cells, although according to our voltage dye assays, it did not hyperpolarize U87 cells. However, the cells did significantly express a variety of differentiation markers after 6 days of treatment. The cells treated with NS1643 and TMZ also showed, similar to the other NS1643 containing treatments, an increase in lysosomal and cytoplasmic pH, an increase in p27^kip1^, and a significant decrease in the YAP nuclear to cytoplasmic ratio. It is possible that the voltage dye did not correctly report the membrane potential with this combination due to an interaction with the dye molecule itself, as has been reported for other compounds [155,156].

In agreement with our results, NS1643 has also been shown to induce senescence in melanoma and breast cancer [55,131,157]. This senescent phenotype is thought to occur through elevated internal calcium levels (a response to hyperpolarization), which trigger the activation of calcineurin, which in turn dephosphorylates NFAT and results in its translocation to the nucleus [134]. In the nucleus, it is possible that translocated NFAT and activated calcineurin could be increasing the expression of p21^WAF1/CIP1^, also found caused by NS1643 [134], by a mechanism similar to that found in differentiating keratinocytes where calcineurin increases Sp1/Sp3-dependent transcription and p21 promoter activity in combination with NFAT [158]. That same study performed on keratinocytes showed that calcineurin inactivation resulted in less p27^kip1^ as well [158]. Interestingly, mitochondrial ROS production has been found to be increased by calcineurin activation in neurons [159], and ROS levels were found elevated in breast cancer cells treated with NS1643 [131]. Elevated ROS levels have been found to decrease the proteasome function [160] and could possibly increase p27^kip1^ levels through decreased ubiquitination and degradation [161]. Currently, no publications have shown the efficacy of NS1643 on glioblastoma or its efficacy in combination with rapamycin, pantoprazole, or TMZ.

Another successful combination included the KCNQ2-5/Kv7.2–7.5 channel opener, and FDAapproved epilepsy treatment, retigabine [87]. Although the application of retigabine alone hyperpolarized NG108-15 cells only slightly and reduced proliferation only marginally by itself, its ability to stop NG108-15 proliferation was increased most significantly with pantoprazole. Pantoprazole, a proton-pump inhibitor, worked well on its own at reducing proliferation in these two cell lines. However, when treatment was removed, the cells immediately re-entered the cell cycle, illustrating that treatment with pantoprazole alone does not arrest the cell cycle long enough to allow for terminal differentiation. When pantoprazole was combined with retigabine, a synergistic effect was achieved that resulted in fewer cells re-entering the cell cycle after treatment was removed. Interestingly we saw a depolarization of the NG108-15 cells when they were treated with the combination, but no change was seen in the membrane potential of the U87 cells.

Pantoprazole alone has been reported to increase the alkalinity of the lysosomes by inhibiting V-ATPase channels [86]. However, we saw no alkalization of the lysosomes and the lysosomal signal was increased. Our results agree with a recent study showing that pantoprazole at neutral pH does not inhibit V-ATPase channels and that it instead increases lysosomal biogenesis [120]. Interestingly, that same study showed that pantoprazole interfered with proteasome function, which might explain why it worked so well as a combinatorial treatment. Recent studies have shown that proteasome dysfunction along with an increase in p27^Kip1^ leads to senescence and endoreplication, resulting in cells with large nuclei [162]. In addition, the loss of PTEN in U87 (ATCC) cells results in cells that preferentially senesce in response to stress [163]. The presence of endoreplication in strongly senescent cells was evidenced by the size of the nuclei in both cell lines and may explain why the BrdU incorporation results were not as low as the cell count data would suggest they should be. The treatment with pantoprazole alone showed less differentiation and senescence when compared to pantoprazole in combination with retigabine, NS1643, TMZ, or rapamycin in U87 cells. Pantoprazole has been shown to be effective against glioblastoma in vitro [83], but to our knowledge, this is the first study that has tested its efficacy in combination with NS1643, lamotrigine, retigabine, rapamycin, or temozolomide.

The leading GBM treatment, TMZ, was not significantly effective at reducing proliferation in NG108-15 cells. This has also been observed for some GBM cases as well. However, our combination of TMZ with pantoprazole in NG108-15 cells did significantly lower their proliferation as compared to pantoprazole alone, but did not terminally differentiate them, as evidenced by the recovery assay. However, we do note that TMZ by itself was not effective in NG108-15 cells but was significantly effective in U87 cells. This effectiveness in U87 cells might explain why the combination of pantoprazole or NS1643 with TMZ was so effective at reducing proliferation and increasing differentiation markers in these cells and not in the NG108-15 line. It should be noted that these combinations also dramatically increased senescence markers in these cells and caused a significant decrease of the YAP nuclear to cytoplasmic ratio.

### 4.5. Drug Concentrations and FDA Status: Paths towards Clinical Use

The drug concentrations used in these studies were chosen to be close to the C_max_ reported for the highest dosages that did not result in unacceptable toxicity. Retigabine, FDA-approved for epilepsy, was used at 10 µM, which is close to the reported upper C_max_ values obtained with a 1200 mg/day dose of about 2250 ng/mL or about 7.4 µM [164]. Blood-brain barrier penetrance is good for retigabine, with free plasma concentrations being about the same as free brain concentrations [165]. Our choice of using a high serum to test our drugs and drug combinations also helps in strengthening the clinical relevance of our treatments, since retigabine also has a high plasma protein binding affinity of about 80% [165]. We do note, however, that the binding of a compound in fetal bovine serum can be different from the binding in human serum [166].

Rapamycin, an FDA-approved drug for immunosuppression, was used at a dose of 100 nM for our immunological studies. This concentration is under the highest dosage of 40 mg/day of a nano-amorphous oral formulation in a fasted state, with a C_max_ of 219 ng/mL or about 239 nM, and toxicity at this level was deemed manageable [167]. Furthermore, rapamycin has been used in a phase 1 clinical trial for glioblastoma and was found to cross the blood-brain barrier effectively [168].

Pantoprazole, an FDA-approved proton pump inhibitor, was used at a dose of 100 µM. The highest dosage of pantoprazole, given for Zollinger-Ellison syndrome is 240 mg/day and results in a C_max_ of 42 mg/L or about 110 µM if the C_max_ is proportional to that given for a 30 mg/day dose (as it is for dosages up to 80 mg/day) [169]. However, pantoprazole has poor blood-brain barrier penetrance of only 2% [170]. Therefore, its use in glioblastoma therapy will have to rely on novel methods for delivery across the blood-brain barrier, of which there are many new strategies being developed [171]. It should be noted, however, that all tests performed on pantoprazole in this study were conducted at a neutral pH, so pantoprazole’s efficacy may increase in an in vivo setting where the tumor microenvironment is more acidic [172].

NS1643, a hERG activator and potentiator of KCNQ2, KCNQ4, and KCNQ2/3 channels, is not currently approved by the FDA. However, it has recently been used in a breast cancer xenograft model in immunodeficient mice and did not show any overt toxicity on the heart or on normal breast epithelial cells [131].

### 4.6. Limitations of the Study

The electrophysiological analysis in NG108 cells showed what immediate changes the compounds had on membrane potential and did not show the changes in potential over time. Pantoprazole did not have a significant effect on the membrane potential of the NG108-15 cells alone; however, the effect of pantoprazole on the negation of the hyperpolarization seen with retigabine, lamotrigine, NS1643, and rapamycin is extremely interesting considering how quickly it occurred. If pantoprazole was merely blocking the function of those other compounds, then it is curious as to why the combination of the compounds would be more effective than pantoprazole alone. More study into the mechanism of this block is needed. In addition, the lack of hyperpolarization in the NG108-15 cells with the combination treatments points to the fact that the hyperpolarization of the membrane potential is not necessarily needed for ion modulating drugs to have an effect. The depolarization of NG108-15 cells seen in the combination of pantoprazole with retigabine or rapamycin could have effects on the ability of the cell cycle to proceed if the needed level of hyperpolarization to proceed through the S phase is not reached, an effect seen specifically in OPCs [173,174,175]. However, this is not the case for the combination of pantoprazole with NS1643 or lamotrigine, which did not have any significant change in resting membrane potential as compared to control in NG108-15 cells.

A more detailed electrophysiological analysis also needs to be undertaken on the U87 cells. In this study, we were only able to find a DiBAC4(3) based analysis of resting membrane potential and may have had some interference with the dye caused by the compounds themselves. The differences in the U87 cell’s membrane potential as compared to the NG108-15 cells after immediate and long-term treatment should also be investigated.

As for the differentiation analysis, it would be interesting to do a more thorough study where multiple markers are stained for in the same sample to see whether markers for more than one lineage are co-expressed in U87 or NG108-15 cells.

## 5. Conclusions

The use of the NG108-15 cell line in high serum conditions for the initial screening of many compounds and combinations gave us a subset of drugs that showed a significant decrease in proliferation in the human U87 glioblastoma cell line treated for six days and showed depressed growth up to four days after treatment was removed. These results suggest that treatment could be given in an intermittent manner. The top combinations were capable of terminally differentiating and causing senescence in NG108-15 cells under normally prohibitive conditions, giving us robust candidates that showed terminal differentiation and senescence in U87 cells. Future studies will test these same compounds on primary human glioblastoma cell lines, patient tumor slices, and in vivo mouse models.

## Figures and Tables

**Table 1:** Compounds with Highest Efficacy on Proliferation and their Mechanism of Action and Status.

Compound	Mechanism	Status
cAMP	Activates variety of ion channels and protein kinases	Cannot be used clinically for GBM [16]
Rapamycin	Inhibits mTOR and induces autophagy	Clinical trial for GBM [81,82]
Retigabine	Opens KCNQ2-5/Kv7.2–7.5 channels	Novel application for GBM
Minoxidil	Opens K(ATP) channels	Novel application for GBM
NS1643	Opens hERG and potentiates KCNQ2-4 channels	Novel application for GBM
Lamotrigine	Blocks voltage gated sodium channels	Novel application for GBM
Pantoprazole	Proton pump inhibitor	Published for GBM [83]
Temozolomide (TMZ)	Alkylates/methylates DNA, induces autophagy, used in GBM treatment	Current standard treatment for GBM [84]

## Data Availability

Primary data not included in the primary or Supplemental Material are available upon request.

## Supplementary Materials

The following supporting information can be downloaded at: https://www.mdpi.com/article/10.3390/cancers14061499/s1, Figure S1: NG108-15 Initial Screen of Compounds Part A; Figure S2: NG108-15 Initial Screen of Compounds Part B; Figure S3: U87 Initial Screen of Compounds; Table S1: Compounds Tested on NG108-15 cells for Effect on Proliferation and their Mechanism of Action and Status [176,177,178,179,180,181,182,183,184,185,186,187,188,189,190,191,192].
