# Supplementary material for: Ion Channel Drugs Suppress Cancer Phenotype in NG108-15 and U87 Cells: Toward Novel Electroceuticals for Glioblastoma
Source: Cancers (Basel). 2022 Mar 15;14(6):1499. doi: 10.3390/cancers14061499 (PMC8946312; doi:10.3390/cancers14061499)
Supplement: Supplementary file 1 [file cancers-14-01499-s001.zip › cancers-1597017-supplementary.pdf]

Supplementary Materials: Ion Channel Drugs Suppress Cancer Phenotype in NG108-15 and U87 Cells: Toward Novel Electroceuticals for Glioblastoma

Juanita Mathews, Franz Kuchling, David Baez-Nieto, Miranda Diberardinis, Jen Q. Pan and Michael Levin

Table S1. Compounds Tested on NG108-15 cells for Effect on Proliferation and their Mechanism of Action and Status.

Compound	Mechanism	Status
**cAMP**	Activates a variety of ion channels and protein kinases	Cannot be used clinically for GBM [16]
**Rapamycin**	Inhibits mTOR and induces autophagy	Clinical trial for GBM [81,82]
**Retigabine**	Opens KCNQ2-5/Kv7.2-7.5 channels	Novel application for GBM
**Minoxidil**	Opens K(ATP) channels	Novel application for GBM
**NS1643**	Opens hERG and potentiates KCNQ2-4 channels	Novel application for GBM
**Gabapentin**	Inhibits voltage-gated calcium channels and reduces HCN4 currents	Novel application for GBM
**Lamotrigine**	Blocks voltage gated sodium channels	Novel application for GBM
**Zolmitriptan**	5-HT1B/D receptor agonist and inhibits high voltage activated Ca^{2+} channels	Novel application for GBM
**Cariporide**	NHE1 inhibitor and acidifies internal pH	Published for GBM [176]
**Topiramate**	Blocks voltage gated sodium and calcium channels and acidifies internal pH	Published for GBM [177]
**Pantoprazole**	Proton pump inhibitor	Published for GBM [83]
**Fenofibrate**	Agonist of the PPARα, depletes ATP, and induces autophagy	Published for GBM [178]
**Acetazolamide**	Carbonic anhydrase inhibitor that acidifies internal pH	Clinical trial for GBM [179]
**Quercetin**	Flavonoid that induces autophagy	Published for GBM [180,181]
**Temozolomide (TMZ)**	Alkylates/methylates DNA, induces autophagy, used in GBM treatment	Current standard treatment for GBM [84]
**Dexamethasone (DEX)**	Corticosteroid, induces autophagy, used in GBM treatment	Currently used to reduce edema in GBM [182]
**ONO-RS-082**	Potassium two pore domain channel (KCNK3) activator	Novel application for GBM
**Topotocan**	Topoisomerase I Inhibitor	Clinical trial for GBM [183–185]
**CKD602**	Topoisomerase I inhibitor	Published for GBM [186]
**z-4-hydroxytamoxifen**	Blocks voltage gated sodium channels and induces autophagy	Clinical trial for GBM [184,187]
**Lansoprazole**	Proton pump inhibitor	Published for GBM [172]
**Monensin**	Sodium ionophore	Published for GBM [188,189]
**Cisplatin**	Binds to purine residues causes DNA damage and cell death	Clinical trial for GBM [190]
**Chlorzoxazone**	Increases activity of large conductance calcium activated potassium (BK(Ca)) channels	Novel application for GBM
**Sodium Butyrate**	Histone deacetylase inhibitor	Published for GBM [191,192]

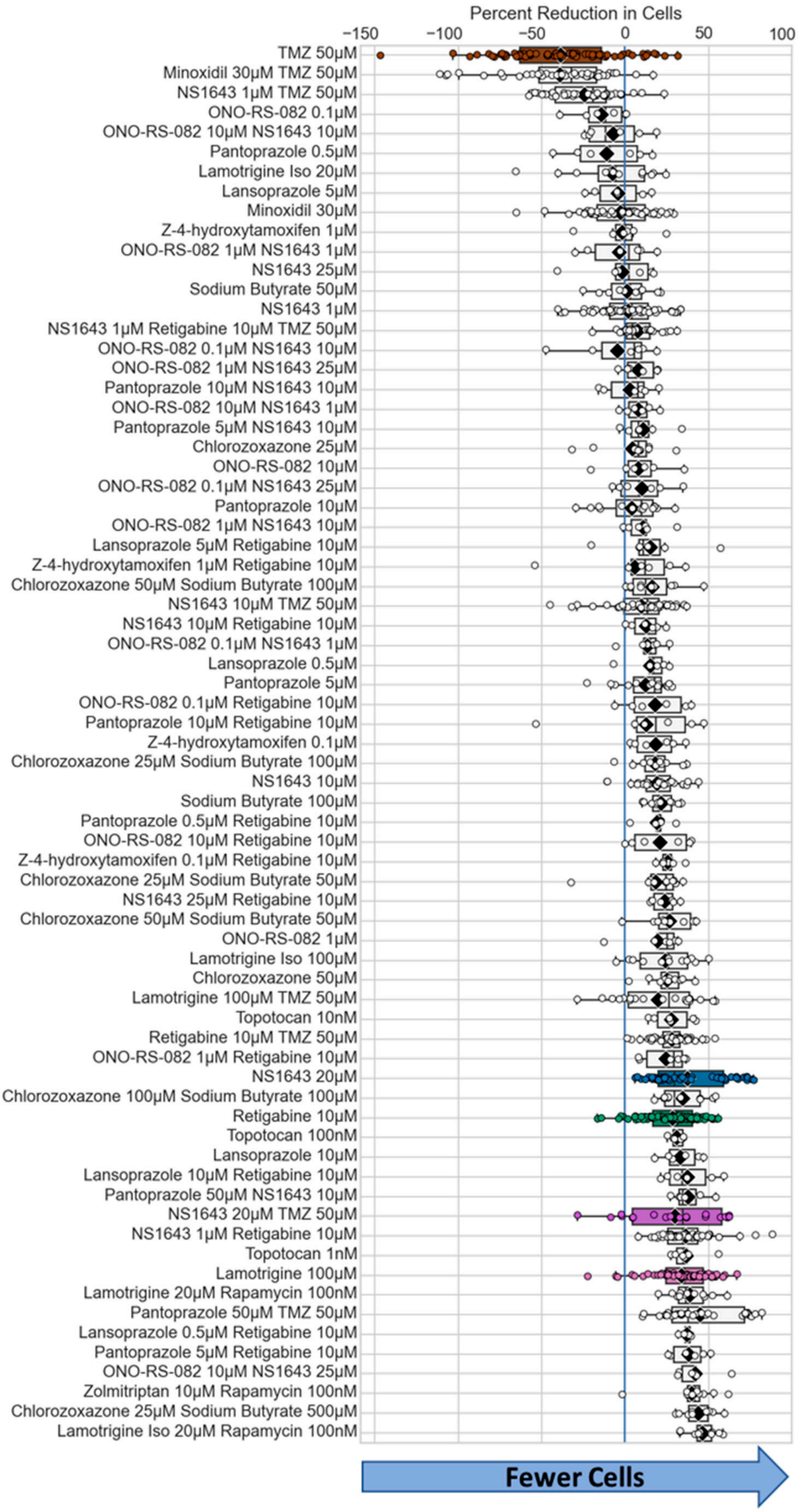

Figure S1. NG108-15 Initial Screen of Compounds Part A. Percent reduction in cells compared to control after 6 days of treatment, *n* >5 technical replicates. Colored plots indicate compounds that were analyzed later in NG108-15 or U87 cells and hues represent concentrations and combinations.

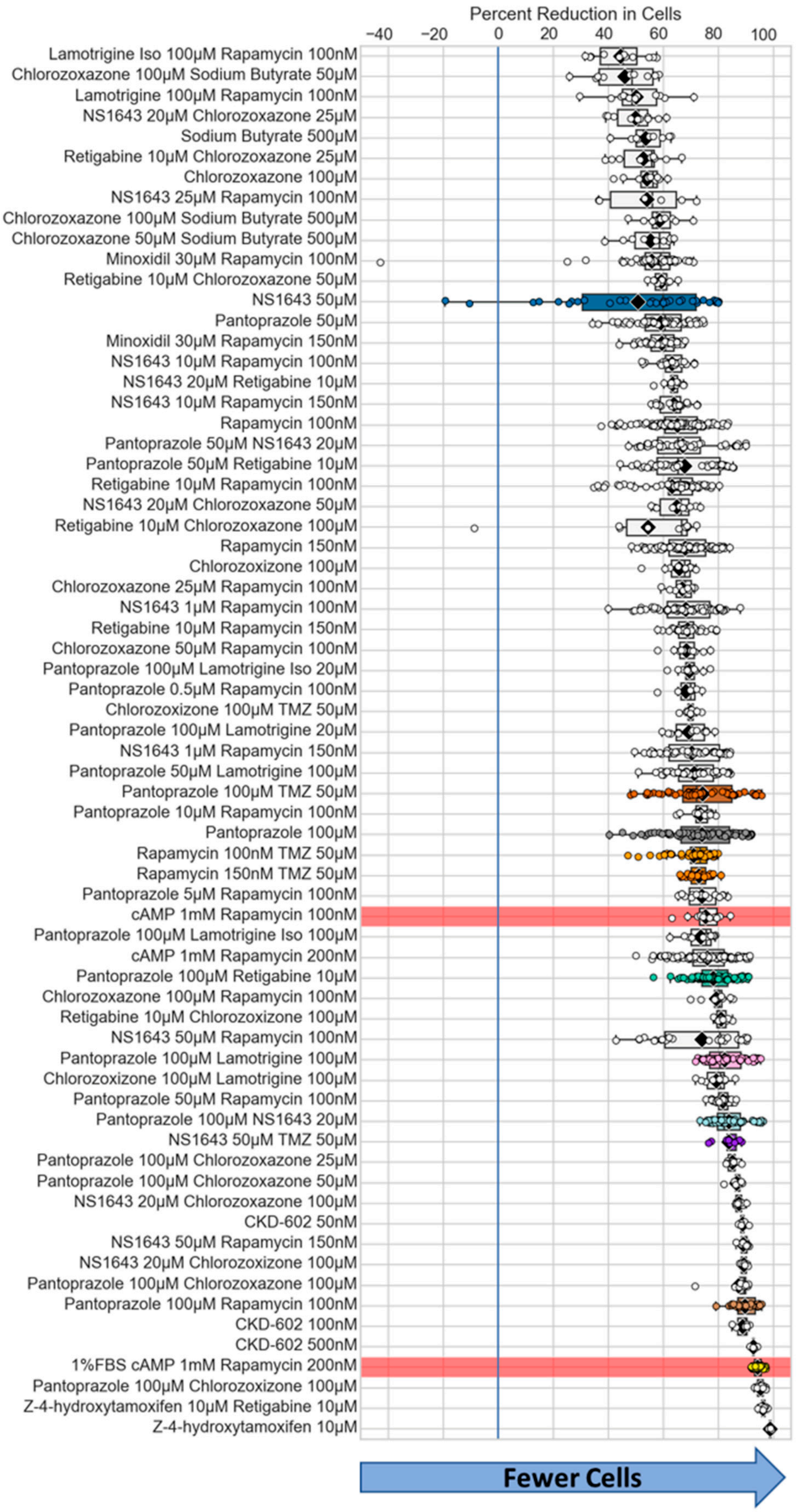

Figure S2. NG108-15 Initial Screen of Compounds Part B. Percent reduction in cells compared to control after 6 days of treatment, *n* >5 technical replicates. Colored plots indicate compounds that were analyzed later in NG108-15 or U87 cells and hues represent concentrations and combinations. Red shaded treatments are positive controls used in later analysis.

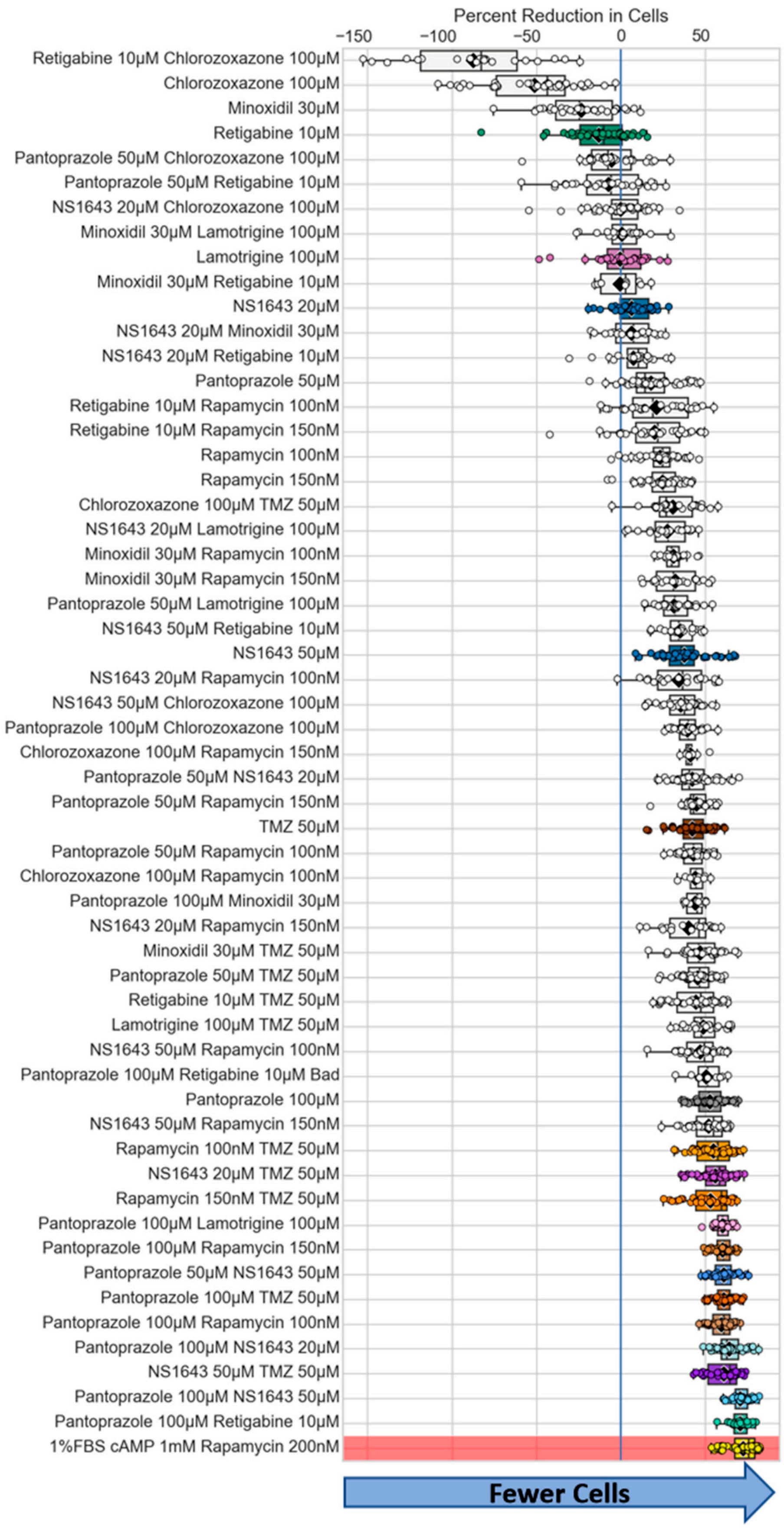

Figure S3. U87 Initial Screen of Compounds. Percent reduction in cells compared to control after 6 days of treatment, *n* >5 technical replicates. Colored plots indicate compounds that were analyzed later in NG108-15 or U87 cells and hues represent concentrations and combinations. Red shaded treatments are positive controls used in later analysis.
